# Supplementary figures and images for: Rapid prediction of key residues for foldability by machine learning model enables the design of highly functional libraries with hyperstable constrained peptide scaffolds
Source: PLoS Comput Biol. 2024 Nov 18;20(11):e1012609. doi: 10.1371/journal.pcbi.1012609 (PMC11611271; doi:10.1371/journal.pcbi.1012609)

## Slide 1
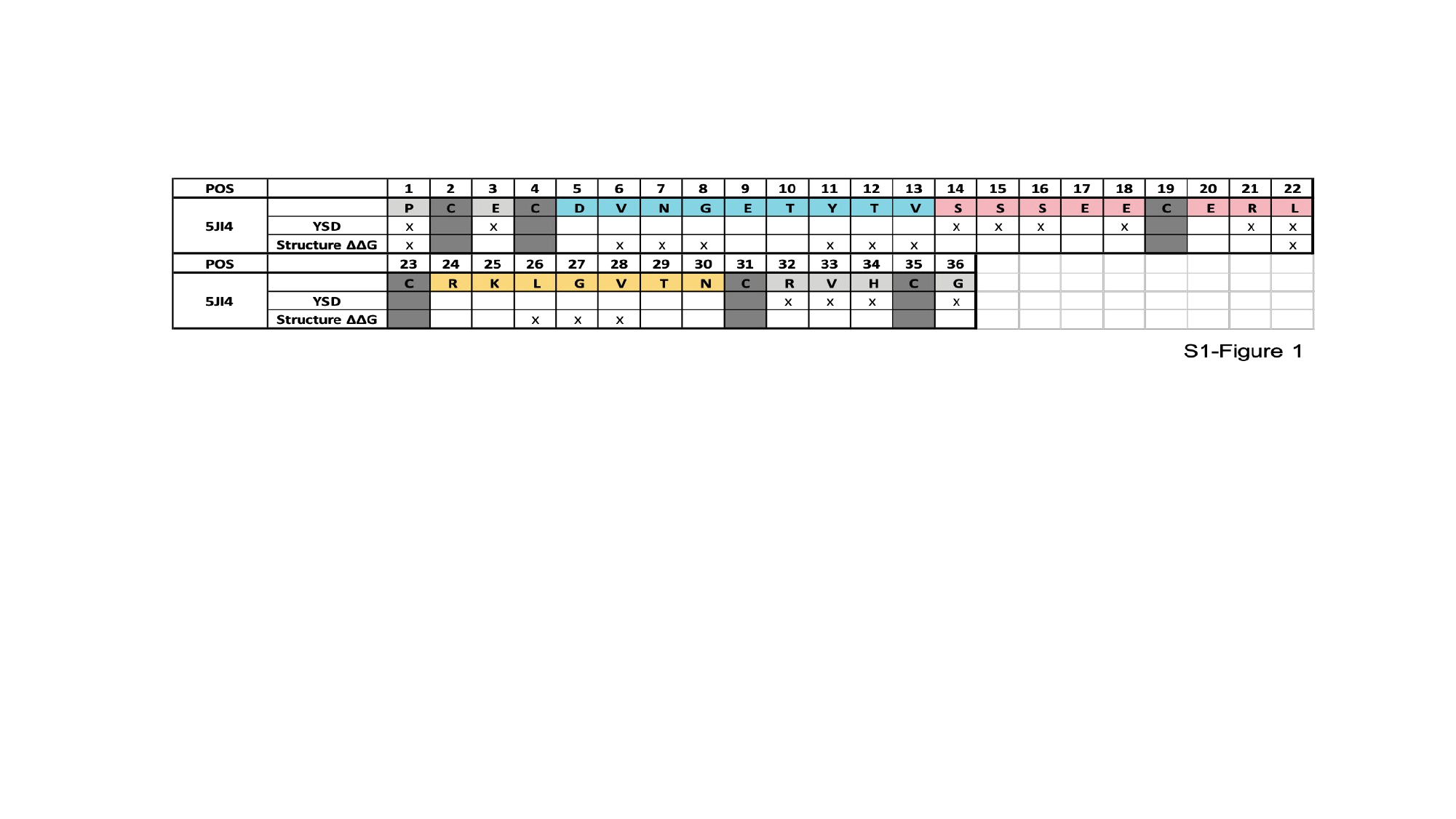

Supplement: S1 Fig — To determine the free energy change upon protein unfolding (ΔΔG), we employed software provided by Cyrus Biotechnology, which utilizes the Rosetta suite for structure prediction. We initiated ΔΔG calculations from the relaxed and energy-minimized structure of the 5JI4 scaffold to ensure the reference conformation was at the lowest possible energy state. We mutate each residue position, with the exception of cysteine residues due to their role in disulfide bond formation, to all amino acids except cysteine. The ΔΔG values were computed for each mutated residue per position. Furthermore, the mean ΔΔG across all mutations per position was calculated, providing an aggregate measure of stability changes attributable to each position. In our analyses, a ΔΔG value falling below the median for the entire scaffold was indicated with an ’X’ in the corresponding table, denoting a residue crucial to folding stability. Conversely, in the context of Yeast Surface Display (YSD), a residue’s Enrichment Score (ES) surpassing the median was marked as ’X’, highlighting its significance. (PPTX) [file pcbi.1012609.s001.pptx]

## Slide 1
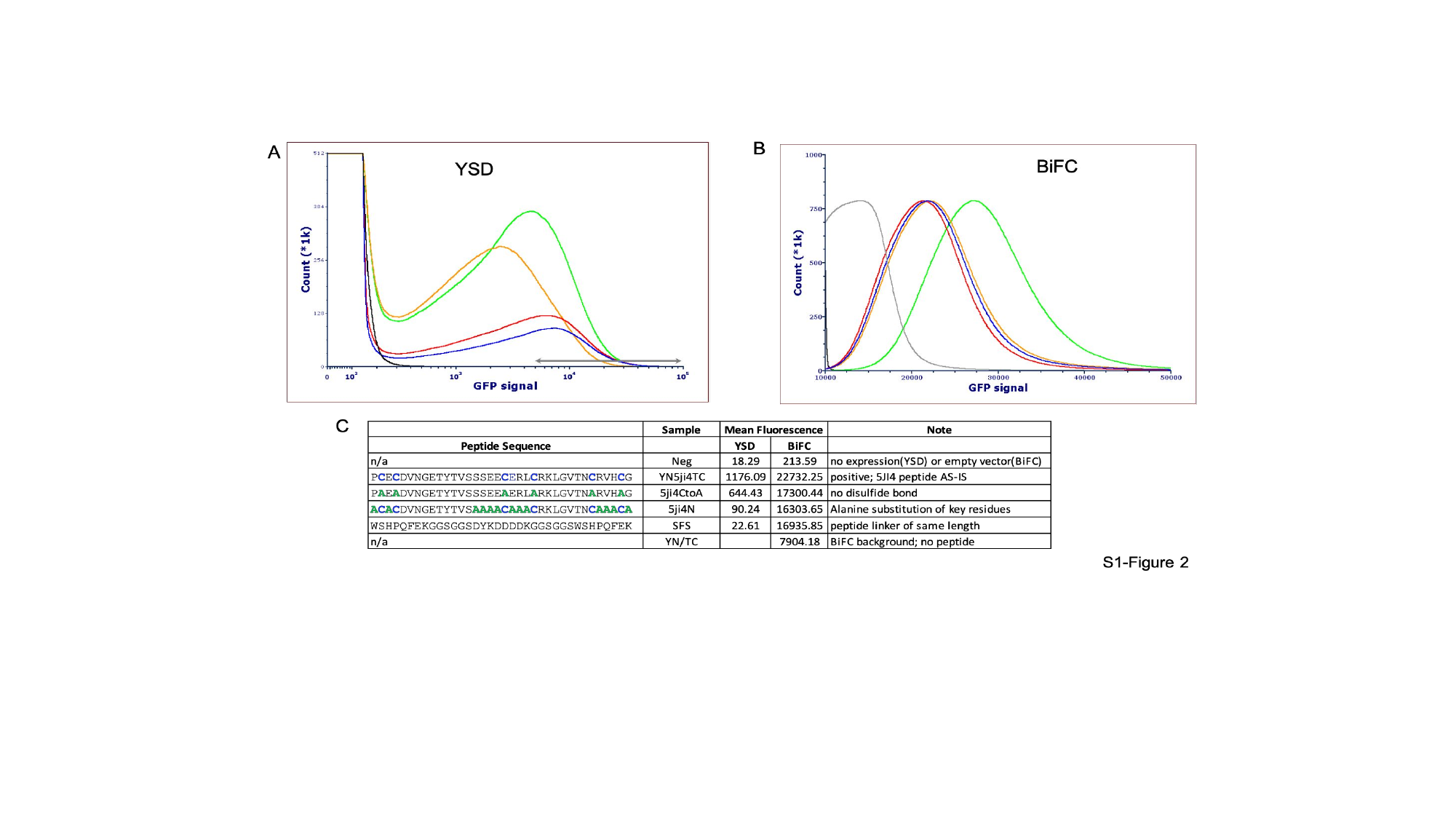

Supplement: S2 Fig — (A) YSD. Yeast cells with no expression (Neg., black line), or expressing peptide 5JI4 (“positive”; green line), 5ji4CtoA (no disulfide bond control; orange line), 5ji4N (Alanine substitution of key residues; red line), or SFS (linker control; blue line) resulted in different distribution pattern in flow cytometry histograms. The grey arrow bar indicated the gating used for FACS sorting. (B) BiFC. E. coli cells harboring the empty vector (Neg., black line), pBAD-YN/TC (“background”; gray line), pBAD-5ji4CtoA (no disulfide bond control; orange line), pBAD-5ji4N (Alanine substitution of key residues; red line), pBAD-SFS (linker control; blue line), and pBAD-YN5ji4TC (“positive”; green line) are well-separated in a flow cytometry histograms. (C) Sequences of control peptides and mean fluorescence for each construct measured using YSD and BiFC. (PPTX) [file pcbi.1012609.s002.pptx]

## Slide 1
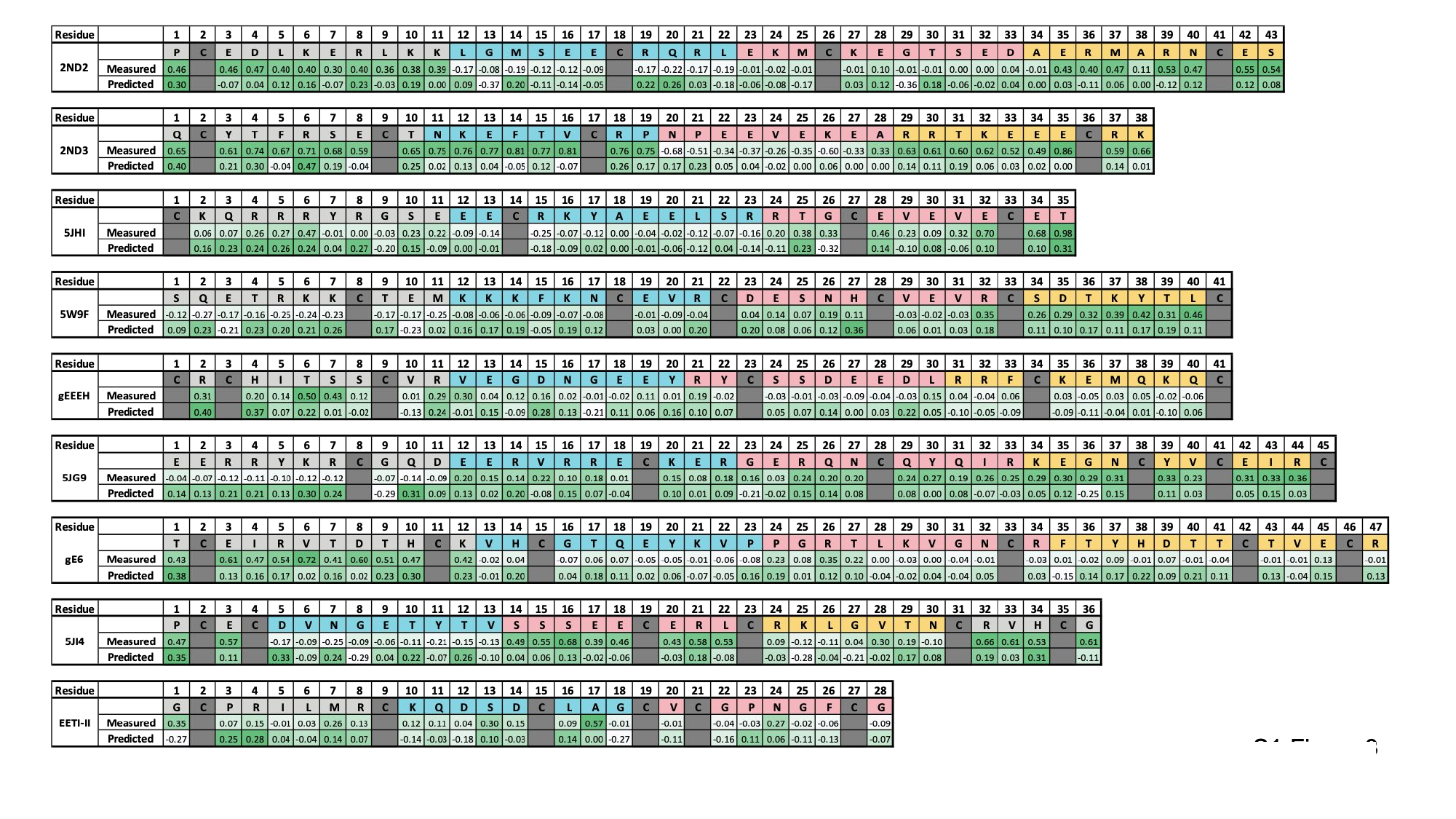

Supplement: S3 Fig — Generation of large datasets from YSD combined with alanine scanning libraries for eight HCP scaffolds and EETI-II scaffold. The same color code as in Fig 1A for library designs and ES heat maps. Showing both measured and predicted ES scores. (PPTX) [file pcbi.1012609.s003.pptx]

## Slide 1
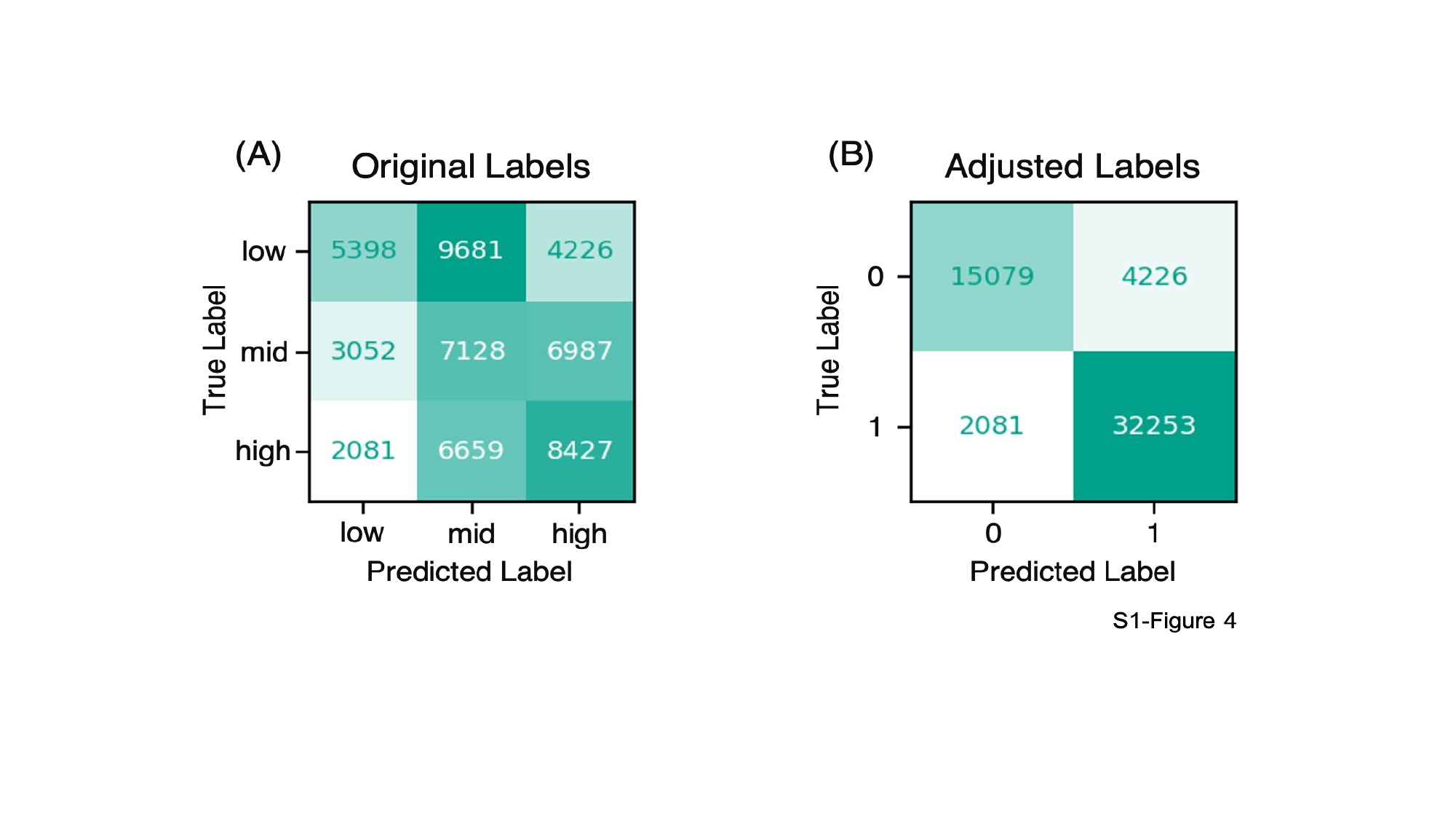

Supplement: S4 Fig — (PPTX) [file pcbi.1012609.s004.pptx]

## Slide 1
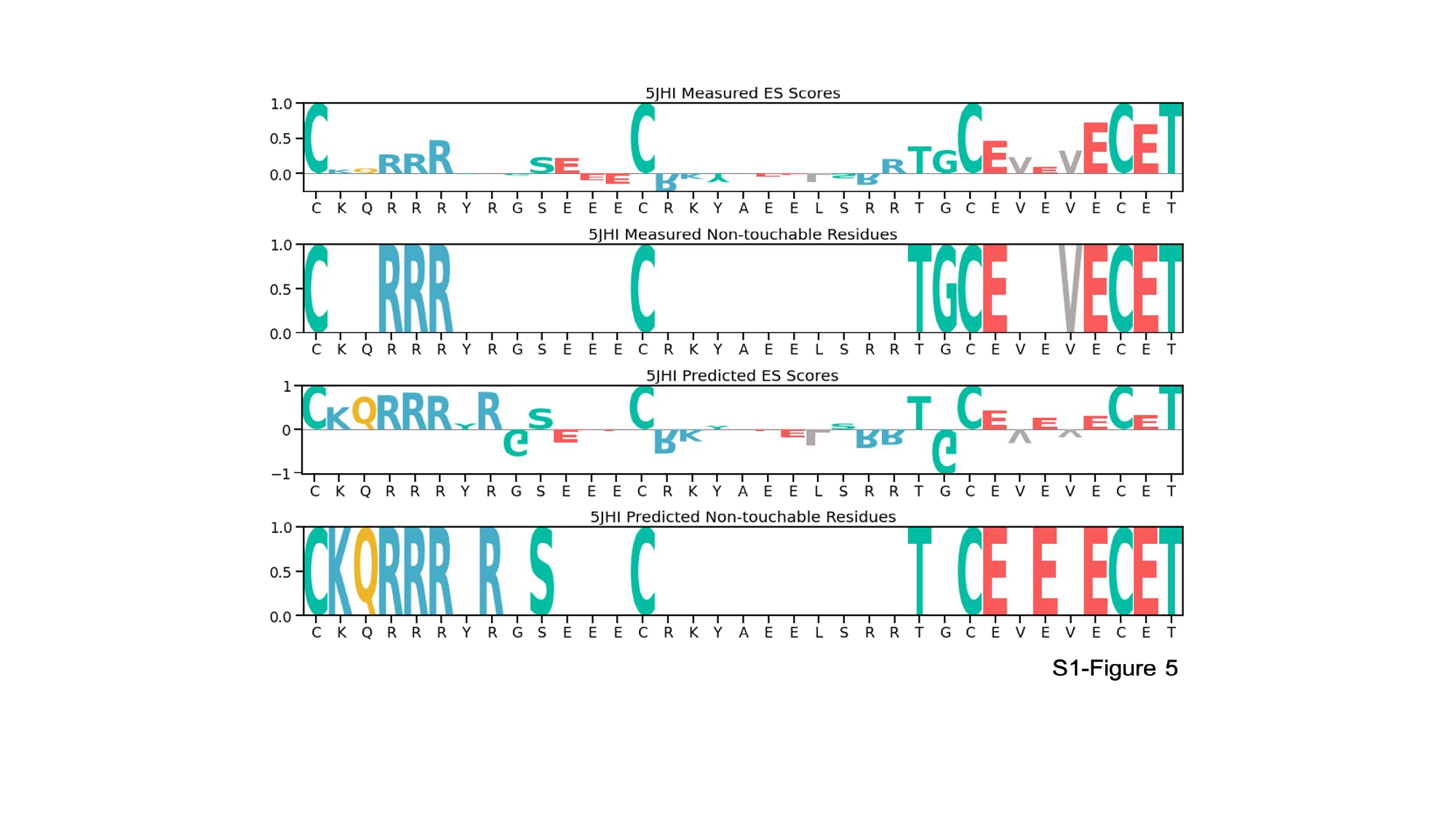

Supplement: S5 Fig — (PPTX) [file pcbi.1012609.s005.pptx]

## Slide 1
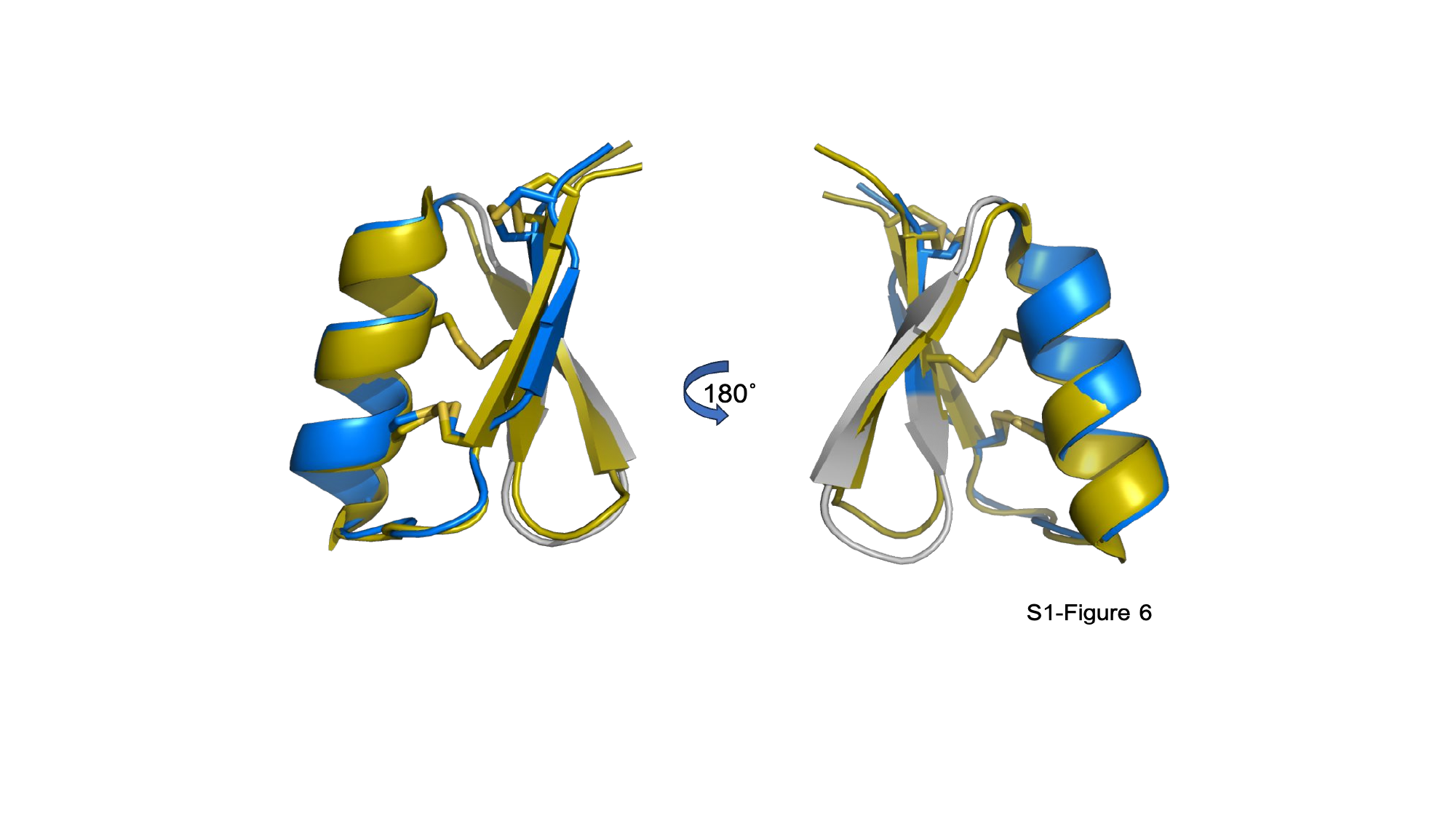

Supplement: S6 Fig — (PPTX) [file pcbi.1012609.s006.pptx]
