# Supplementary material for: Rapid prediction of key residues for foldability by machine learning model enables the design of highly functional libraries with hyperstable constrained peptide scaffolds
Source: PLoS Comput Biol. 2024 Nov 18;20(11):e1012609. doi: 10.1371/journal.pcbi.1012609 (PMC11611271; doi:10.1371/journal.pcbi.1012609)
Supplement: S1 Data — (PPTX) [file pcbi.1012609.s010.pptx]

## Slide 1
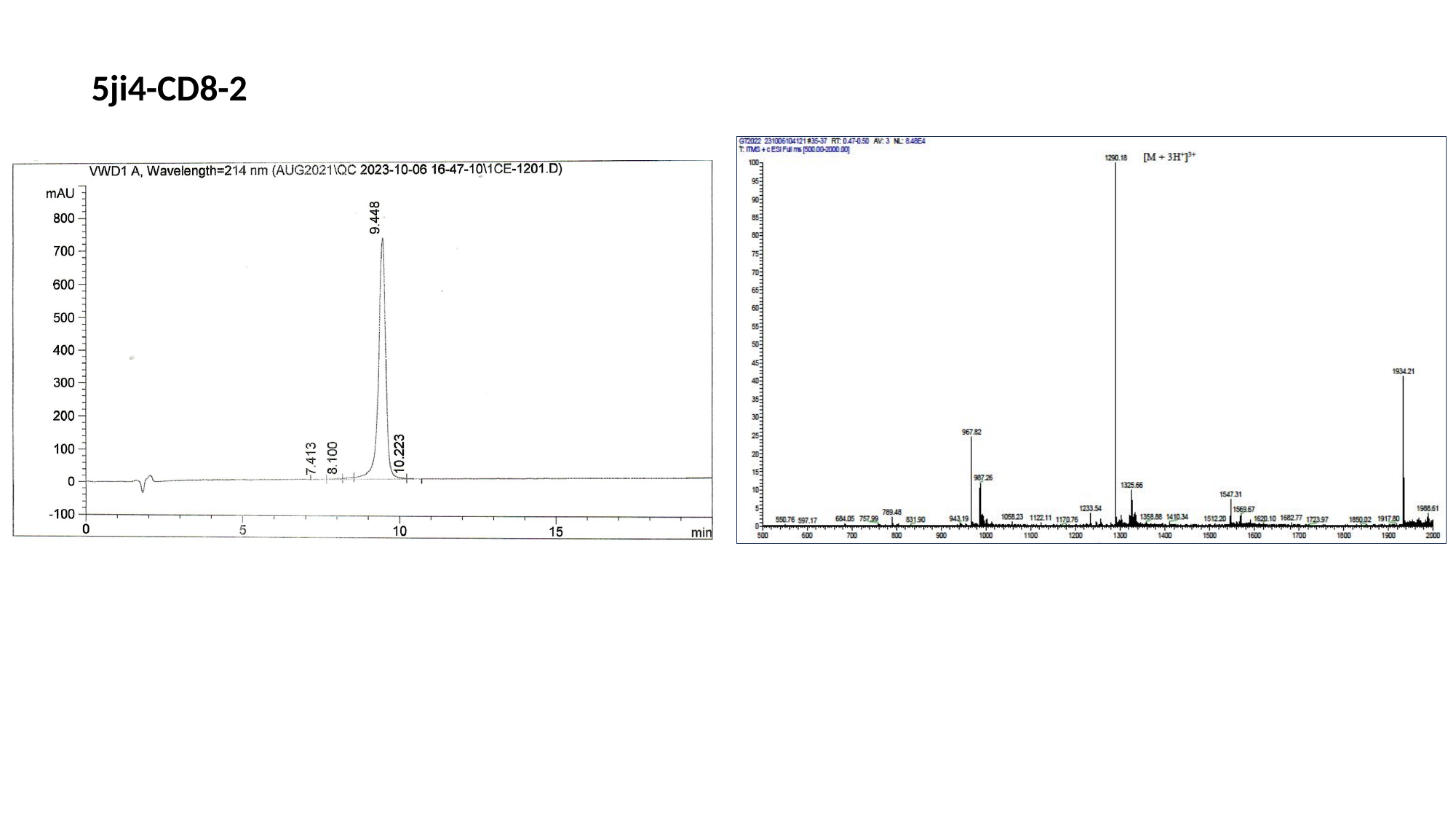

5ji4-CD8-2

## Slide 2
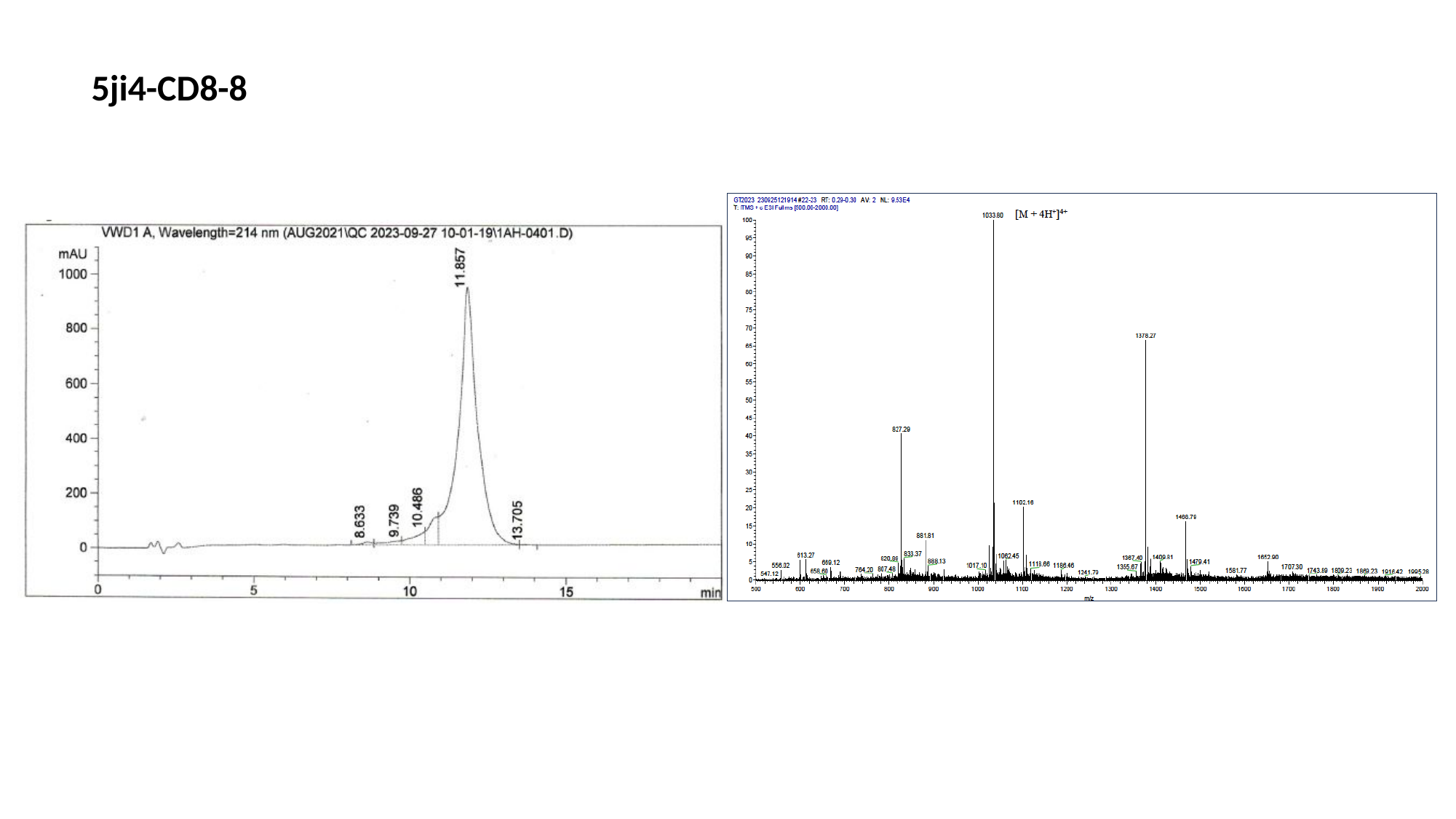

5ji4-CD8-8

## Slide 3
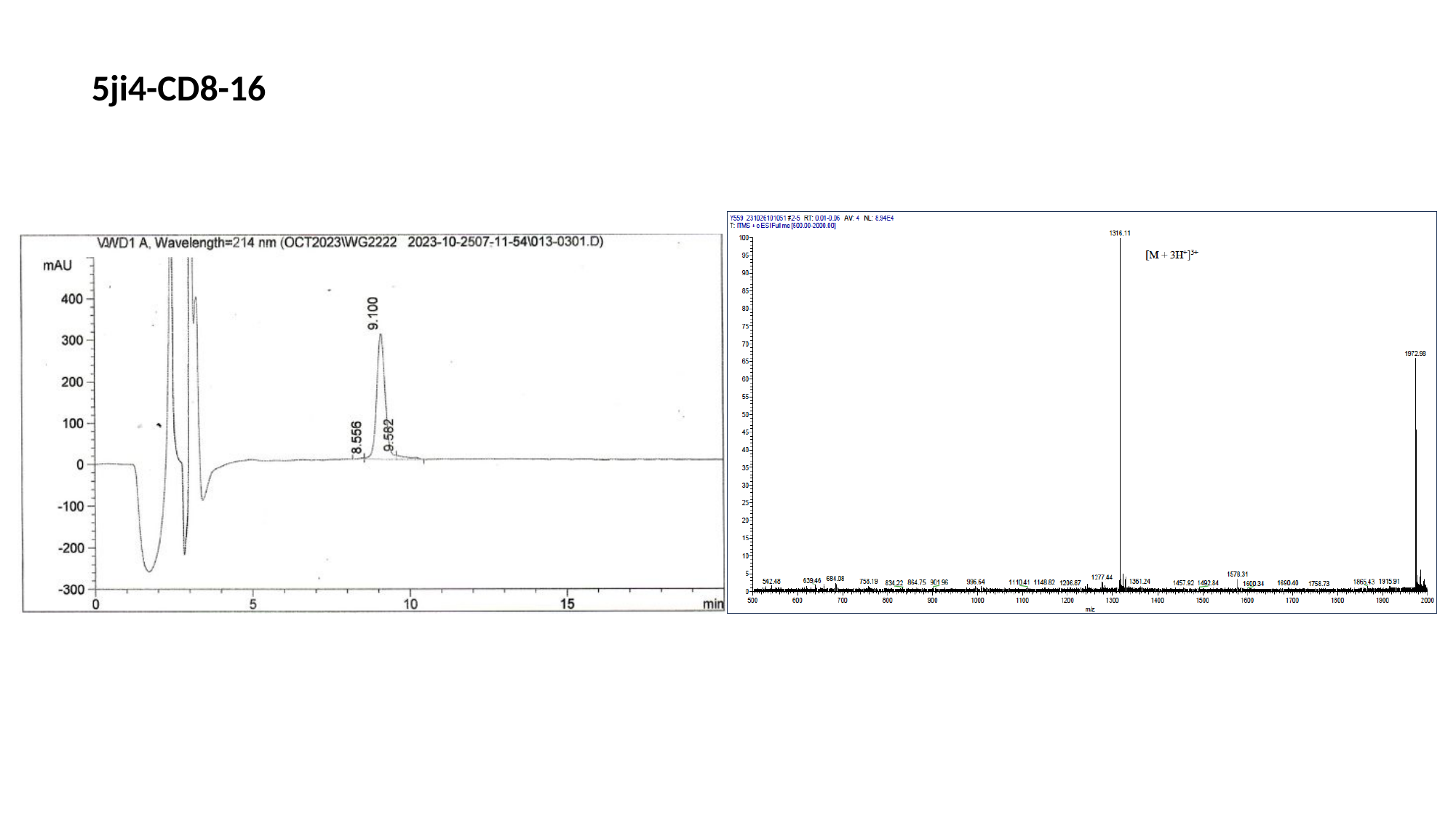

5ji4-CD8-16

## Slide 4
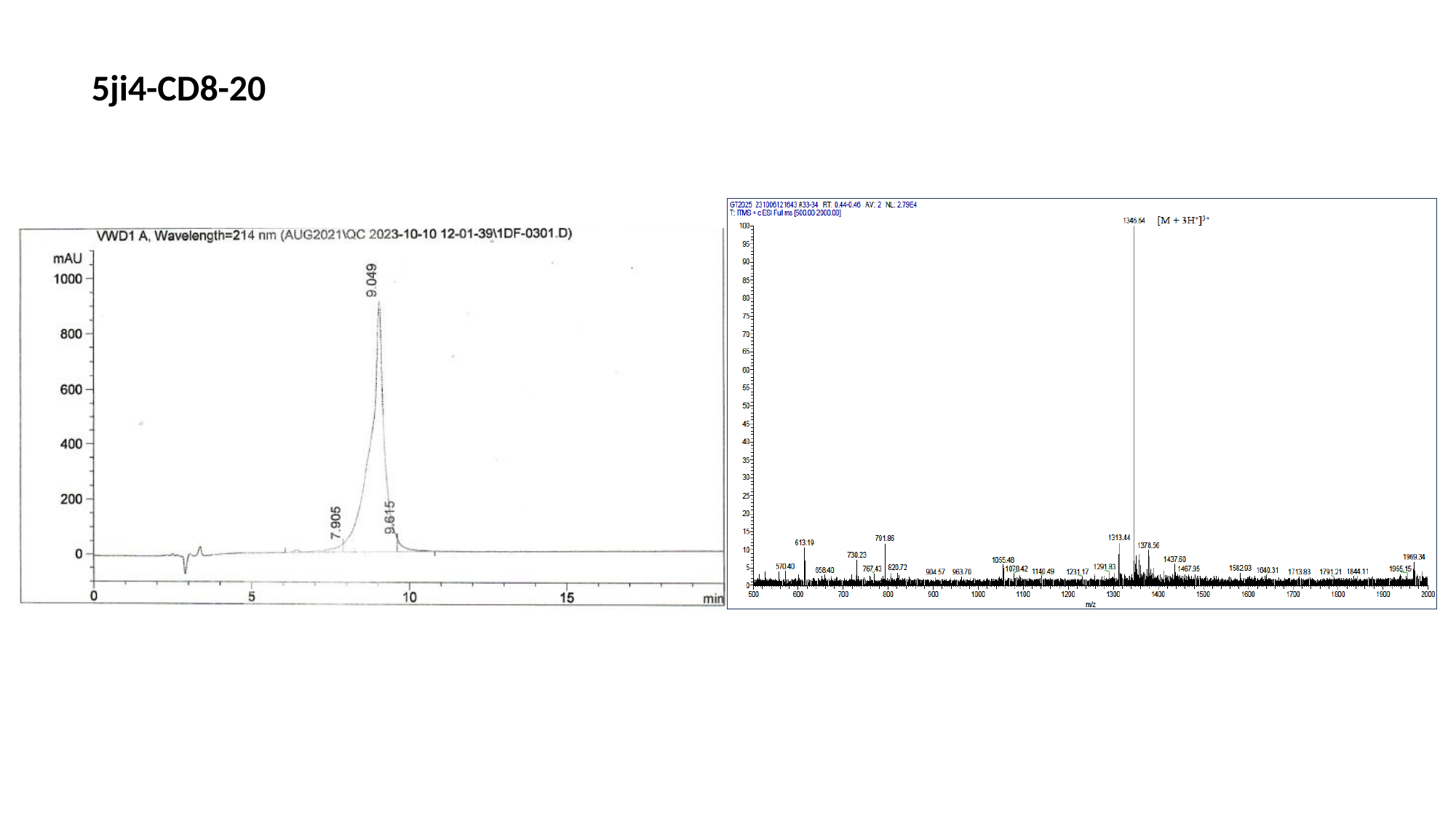

5ji4-CD8-20

## Slide 5
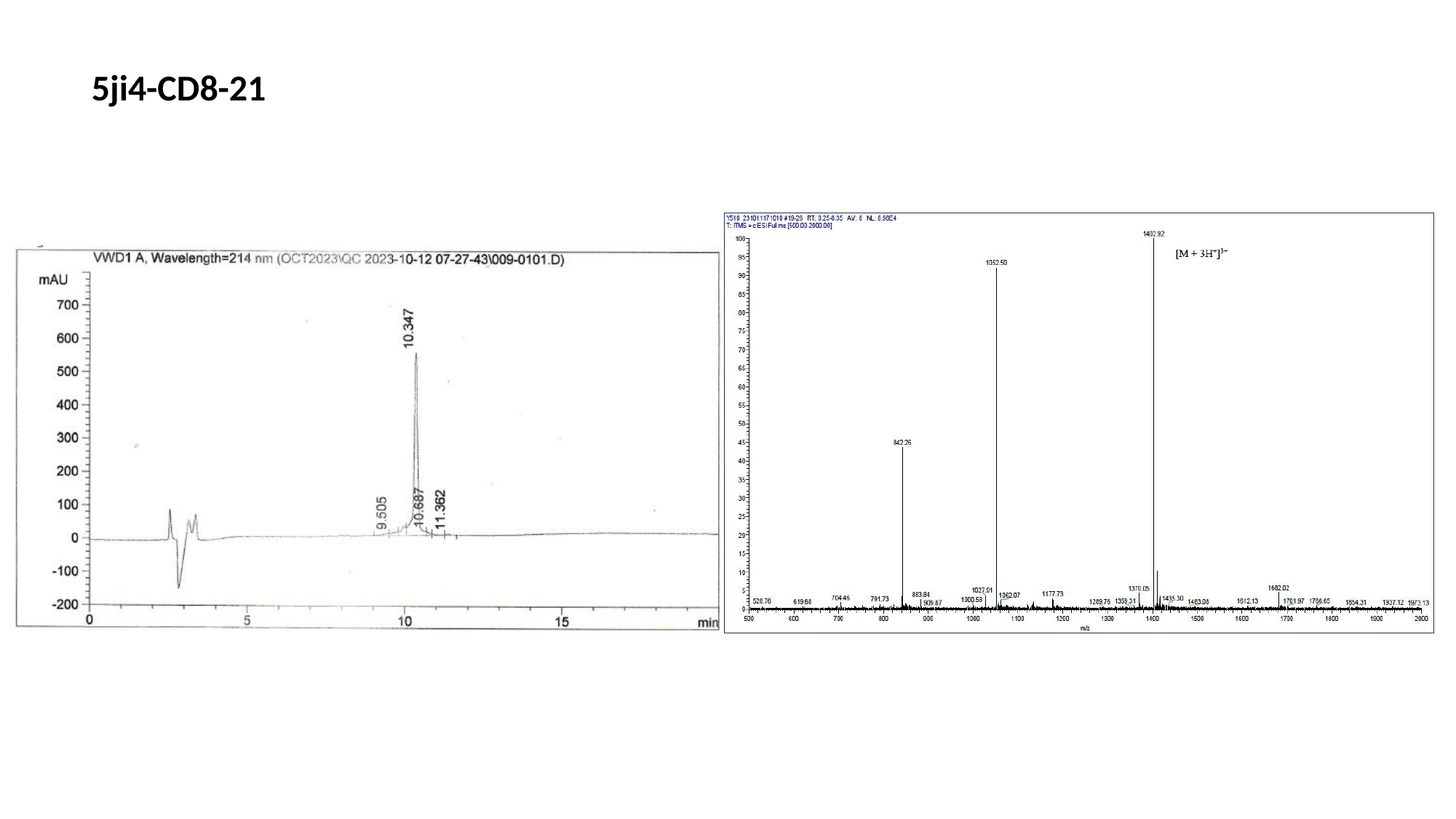

5ji4-CD8-21

## Slide 6
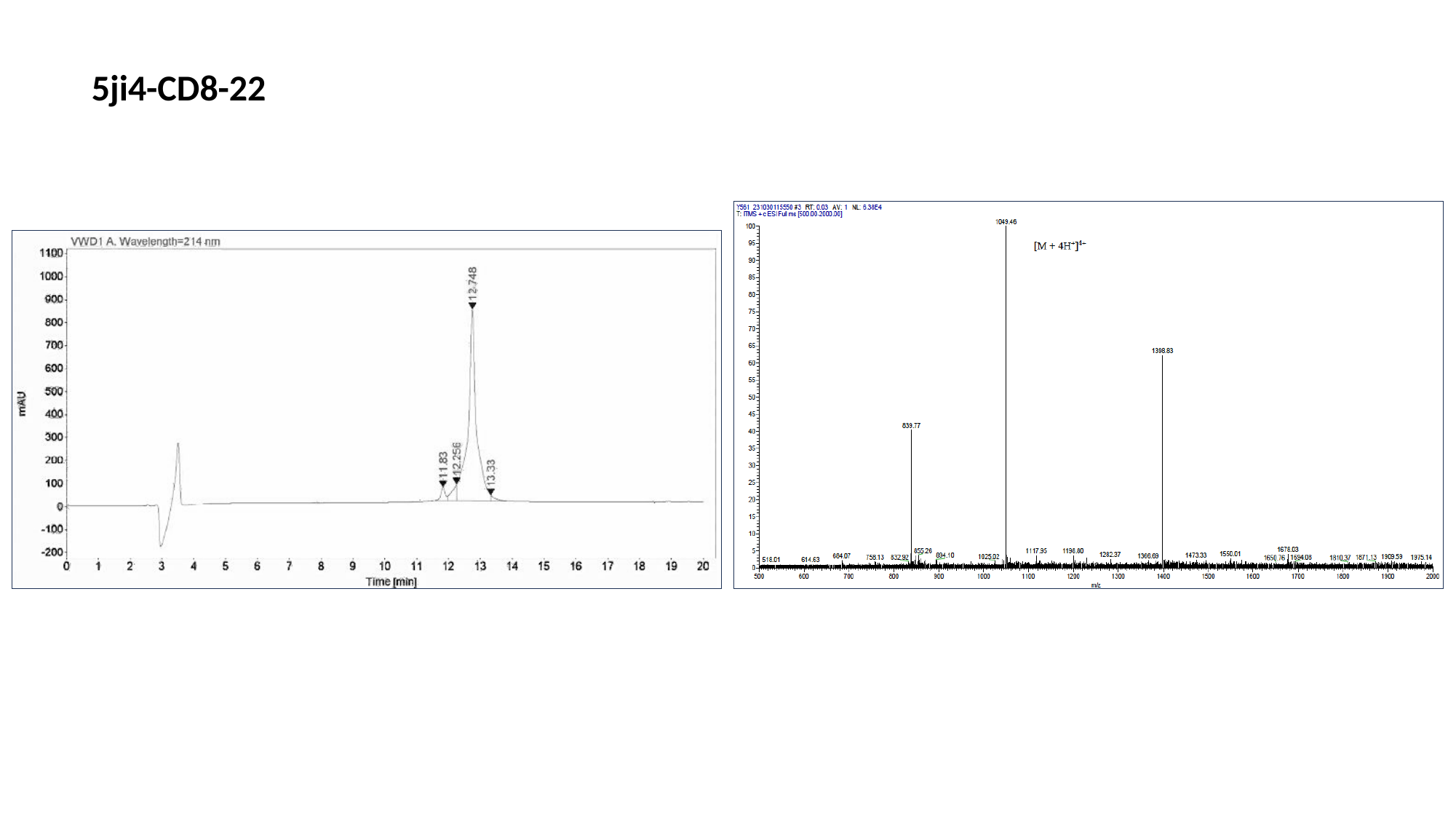

5ji4-CD8-22

## Slide 7
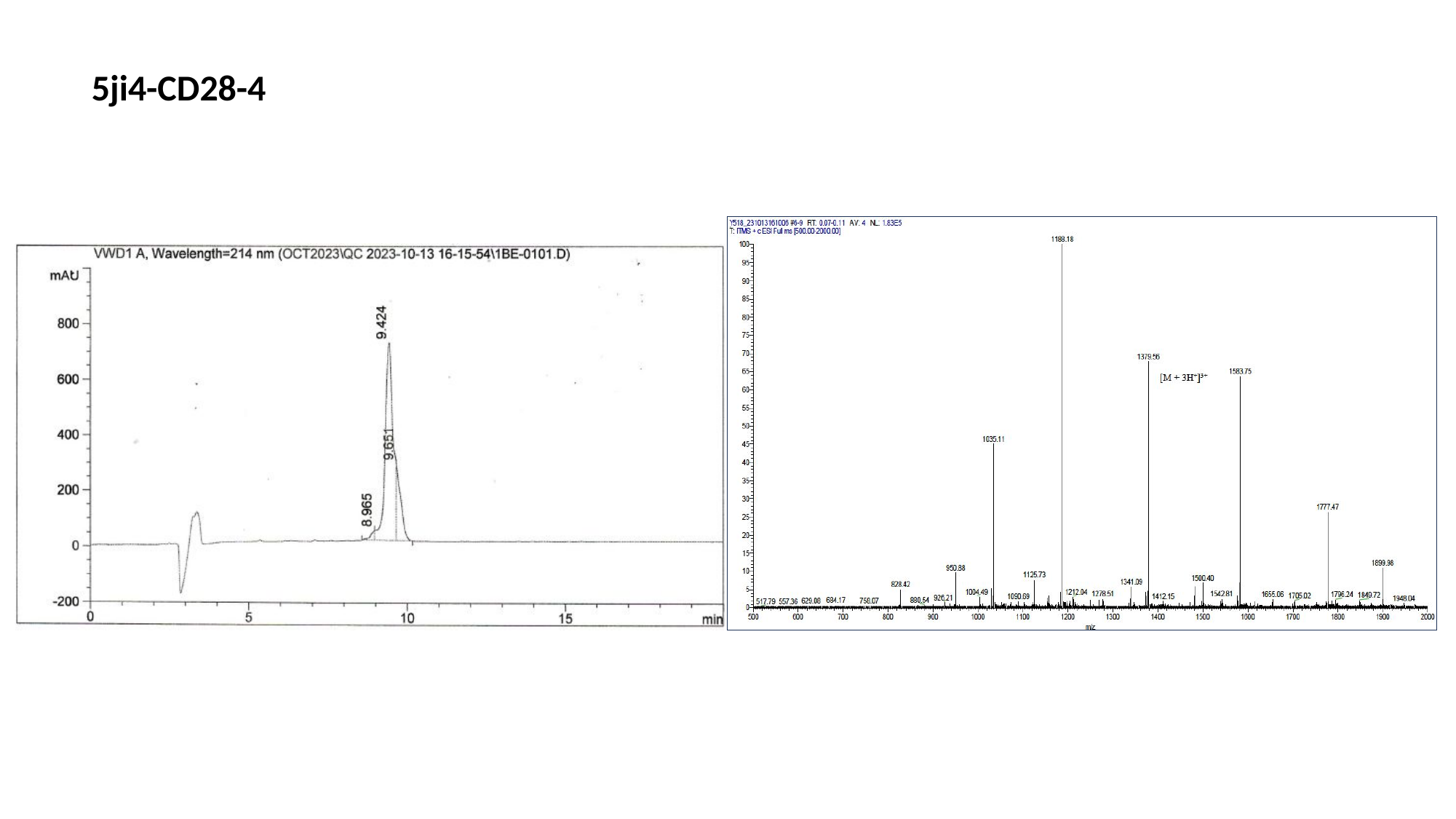

5ji4-CD28-4

## Slide 8
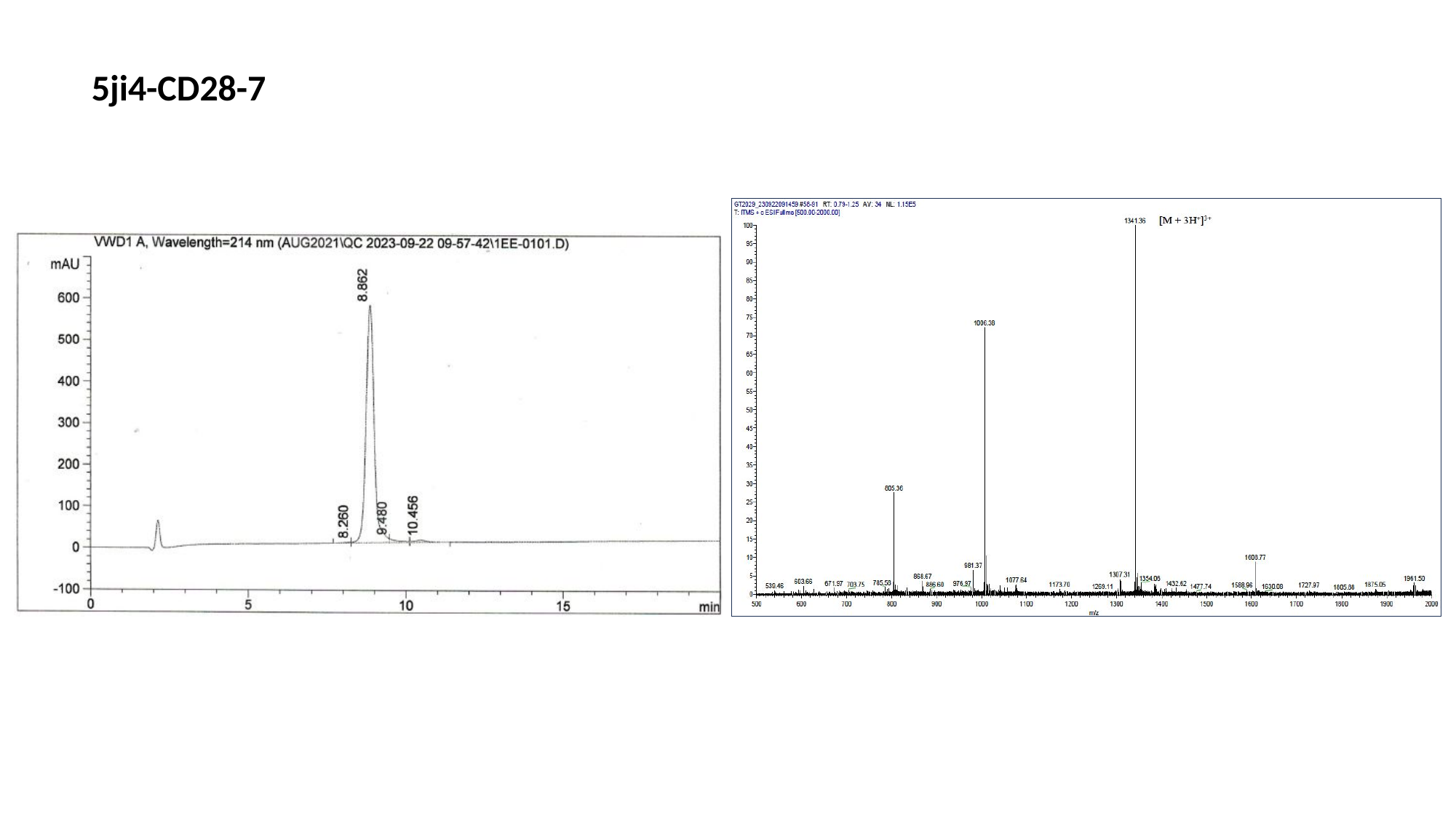

5ji4-CD28-7

## Slide 9
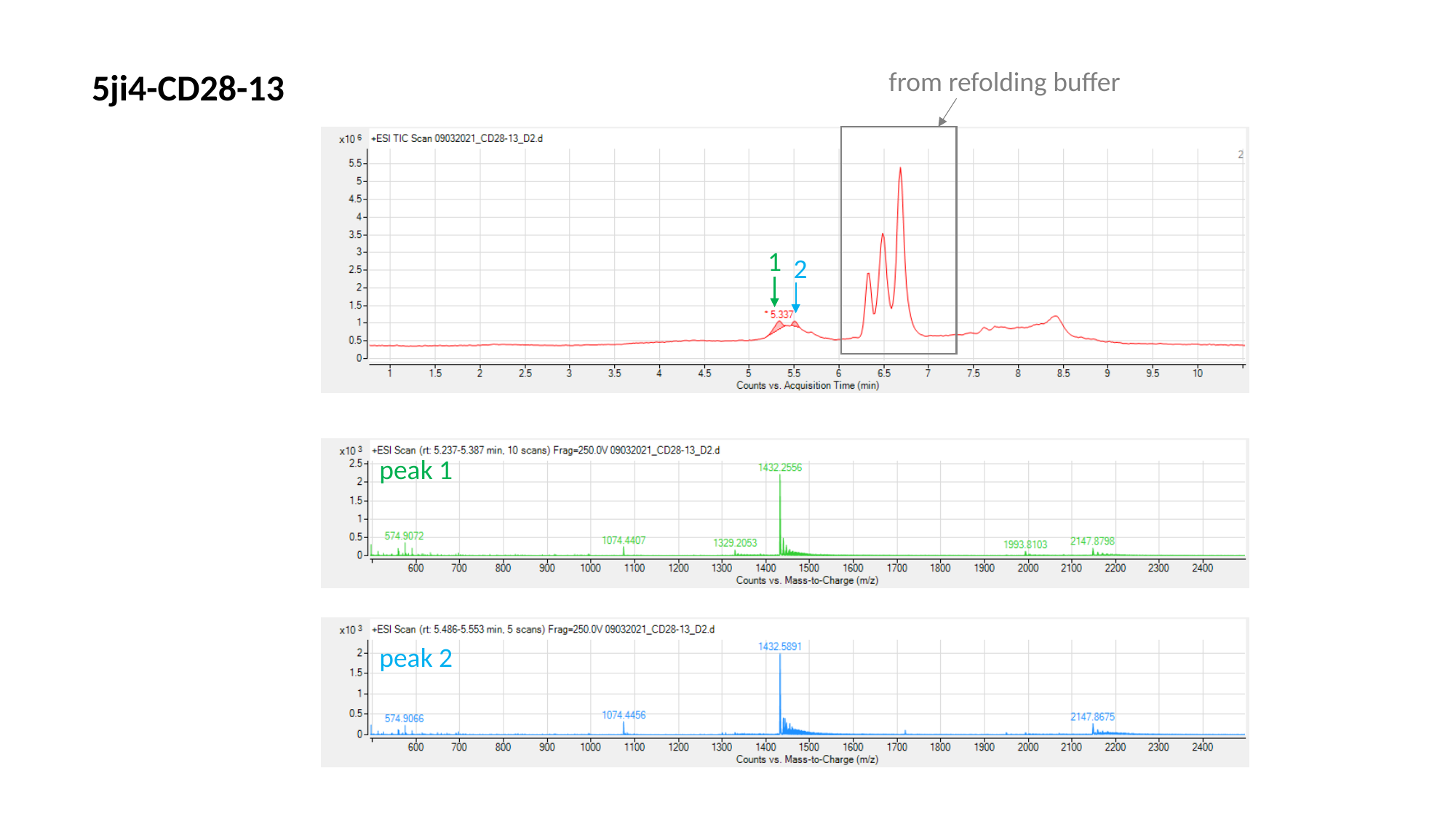

from refolding buffer
1
2
5ji4-CD28-13
peak 1
peak 2

## Slide 10
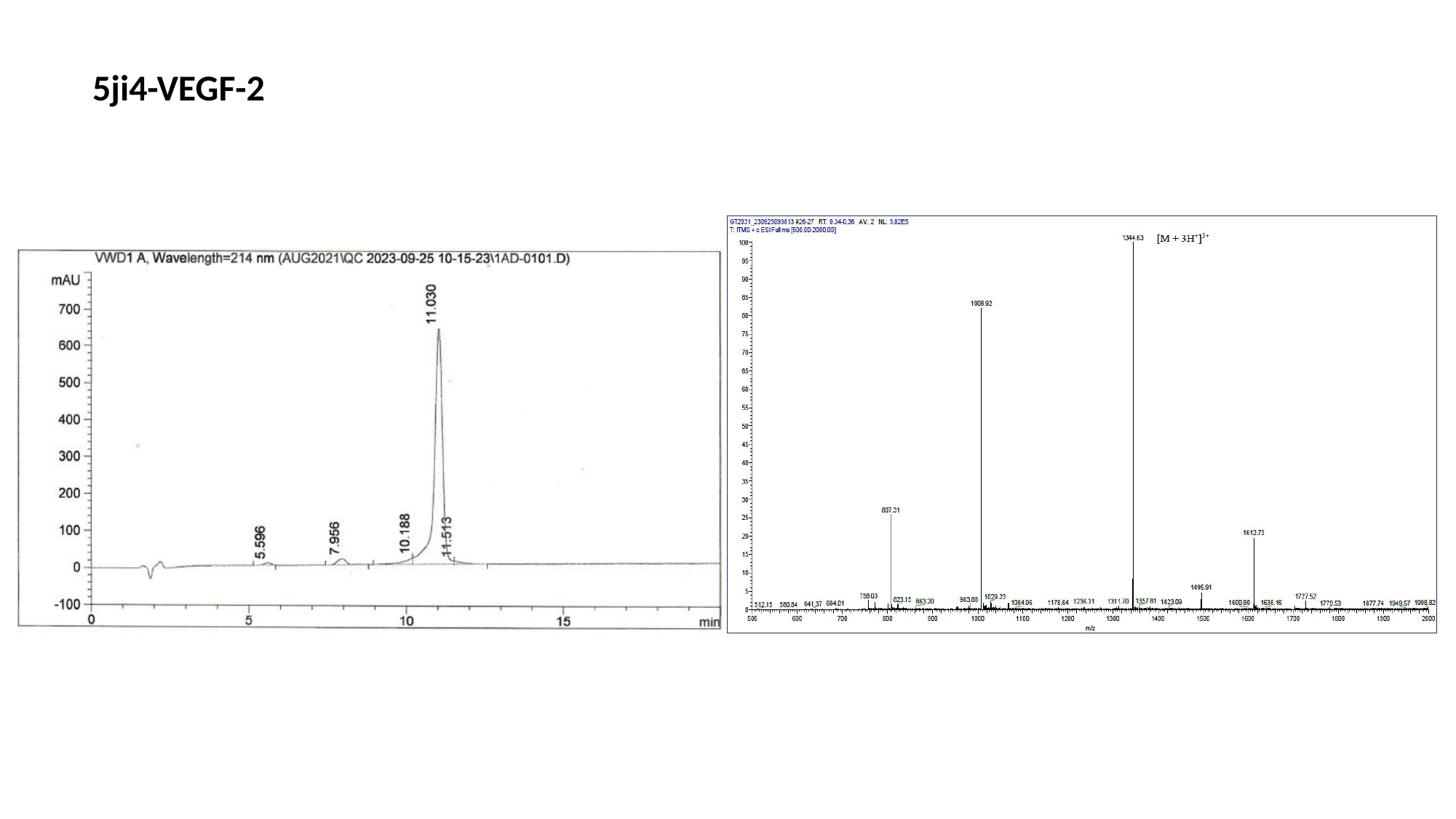

5ji4-VEGF-2

## Slide 11
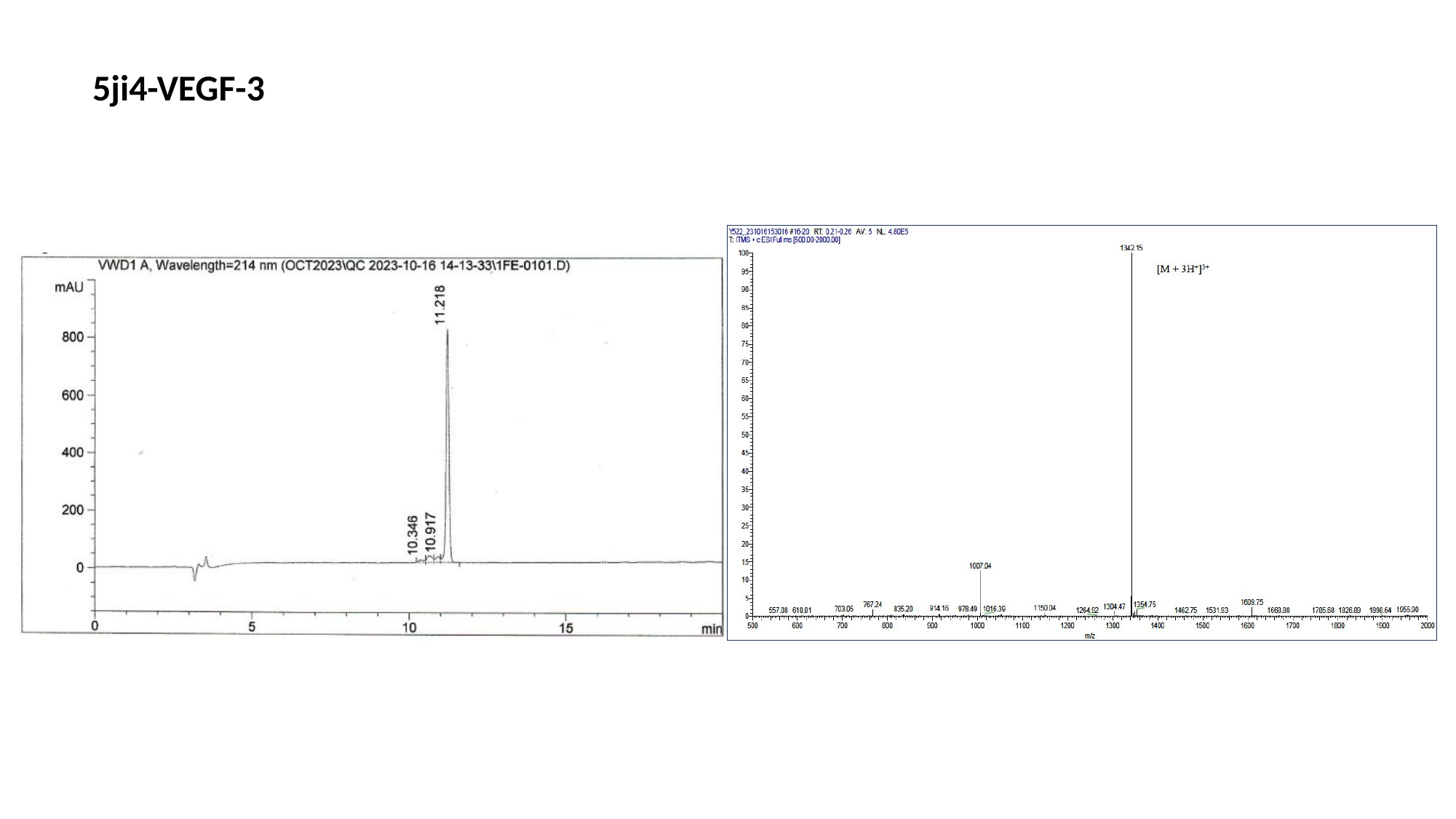

5ji4-VEGF-3

## Slide 12
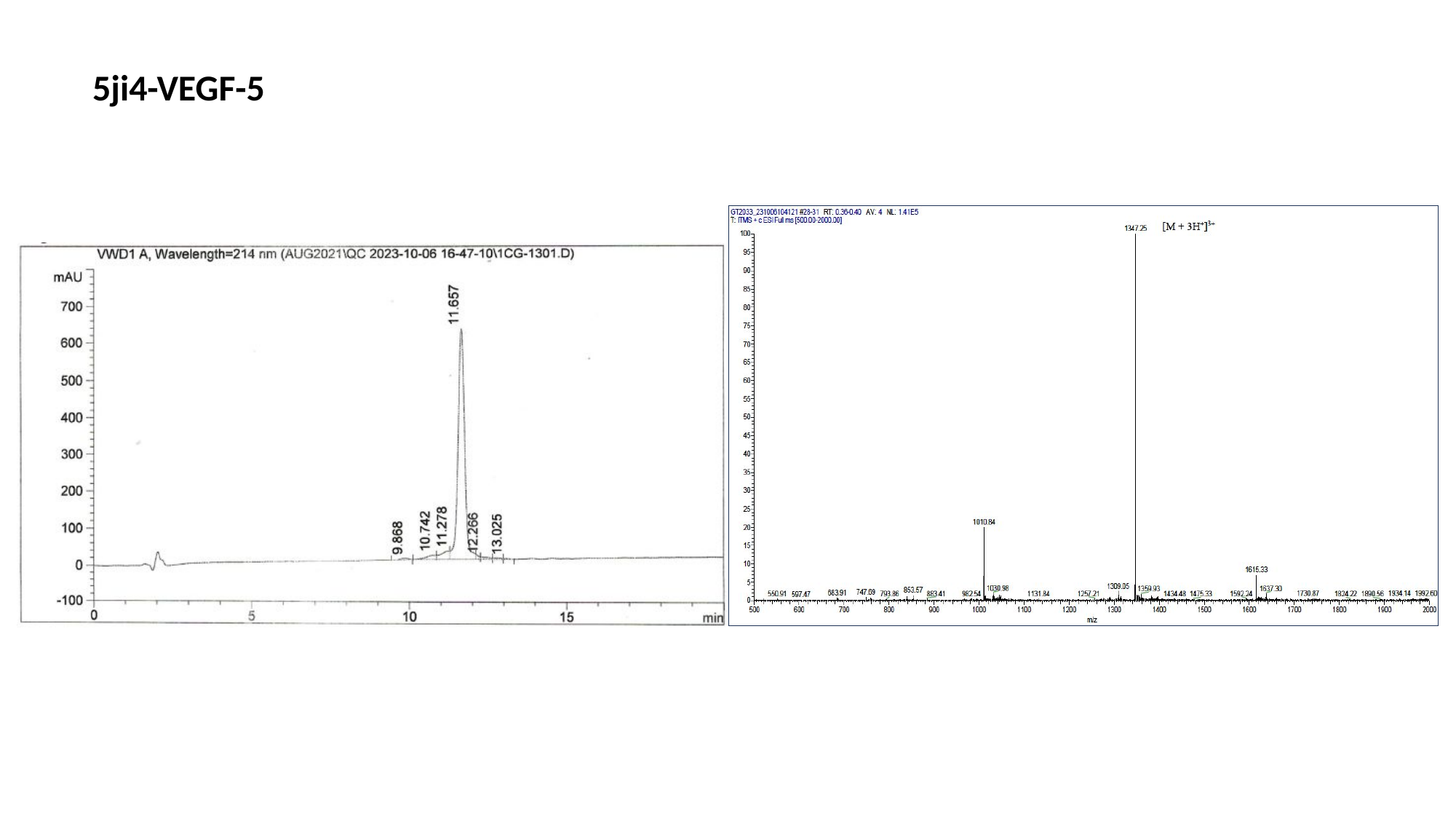

5ji4-VEGF-5

## Slide 13
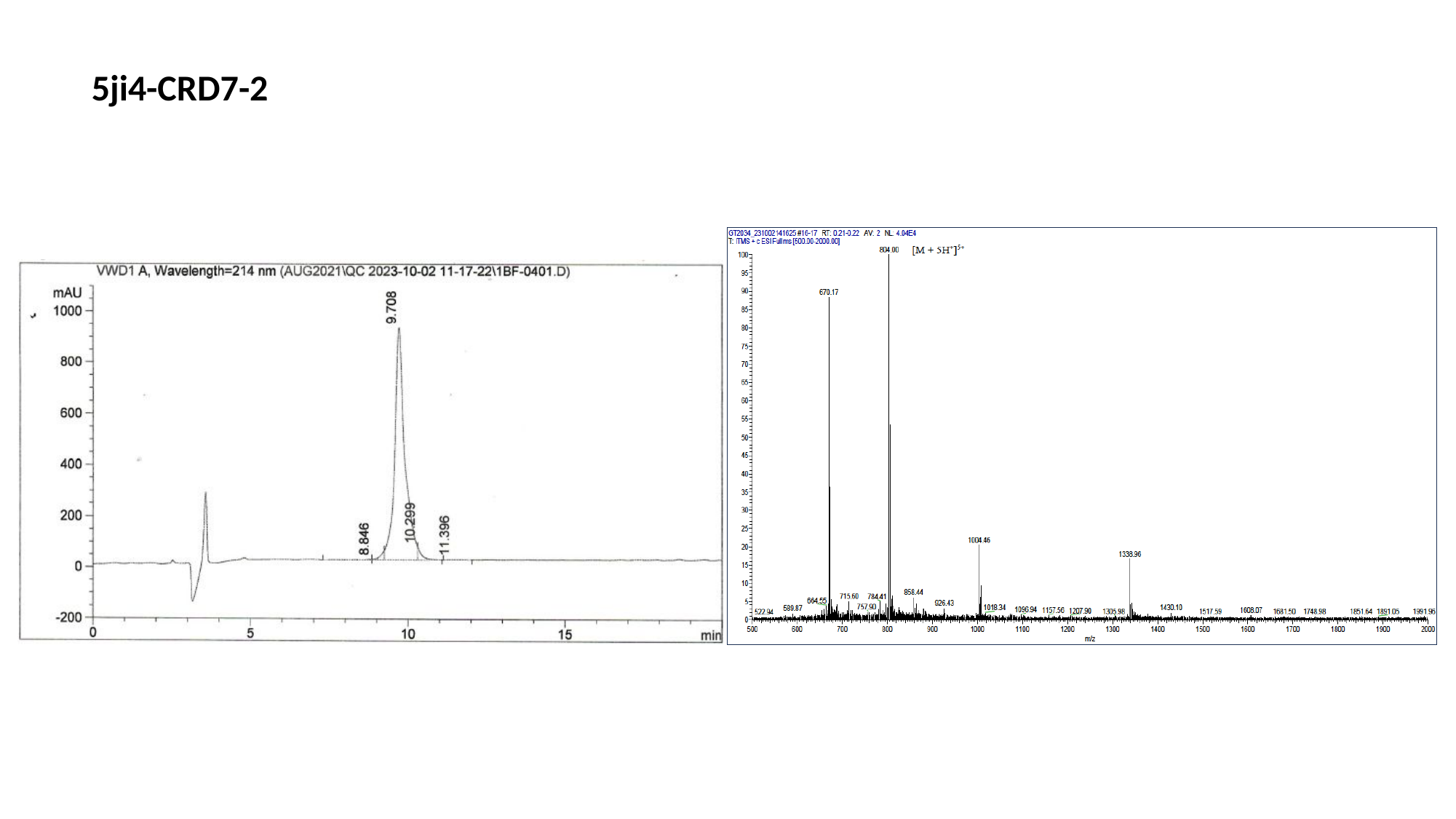

5ji4-CRD7-2

## Slide 14
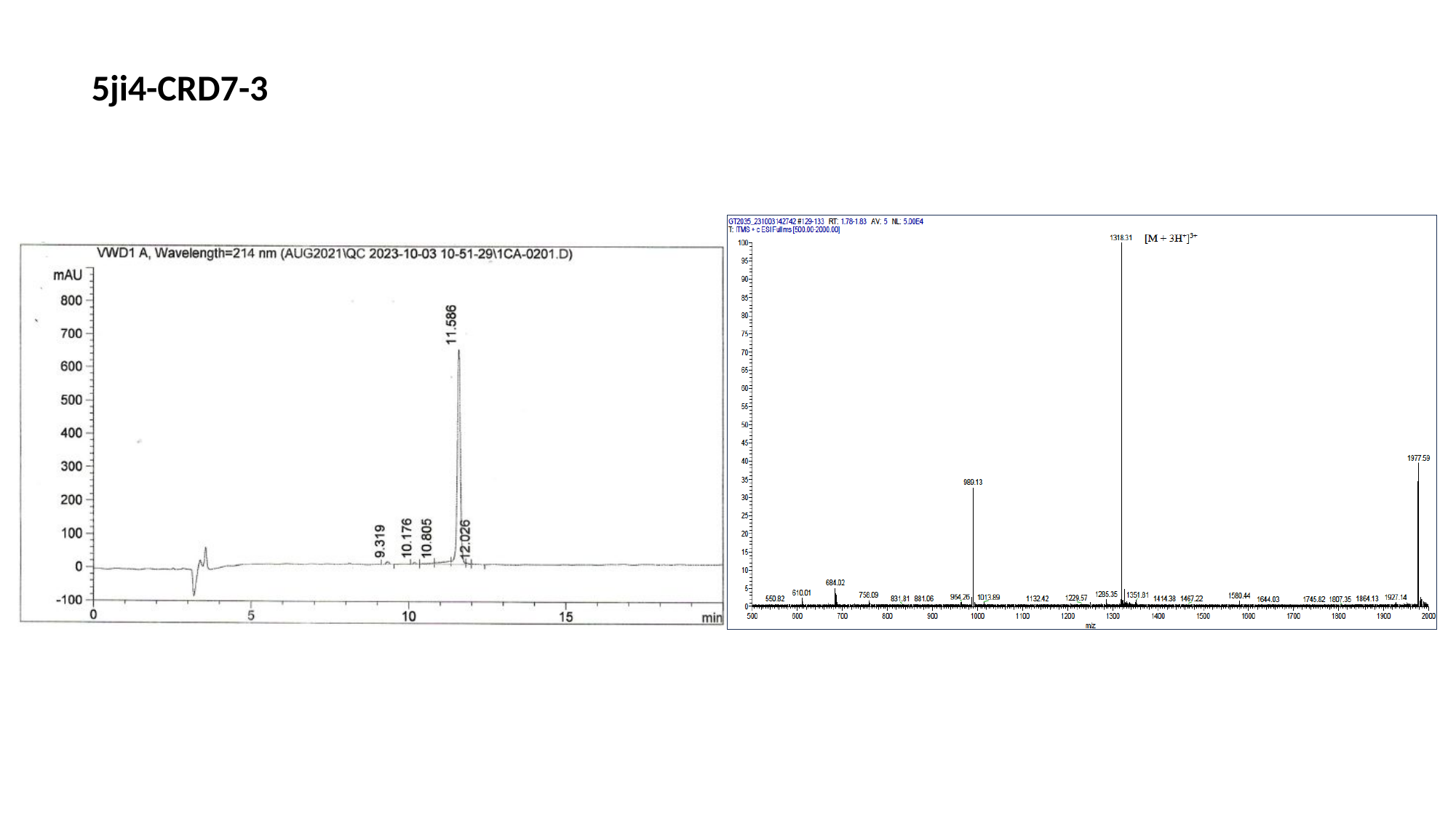

5ji4-CRD7-3

## Slide 15
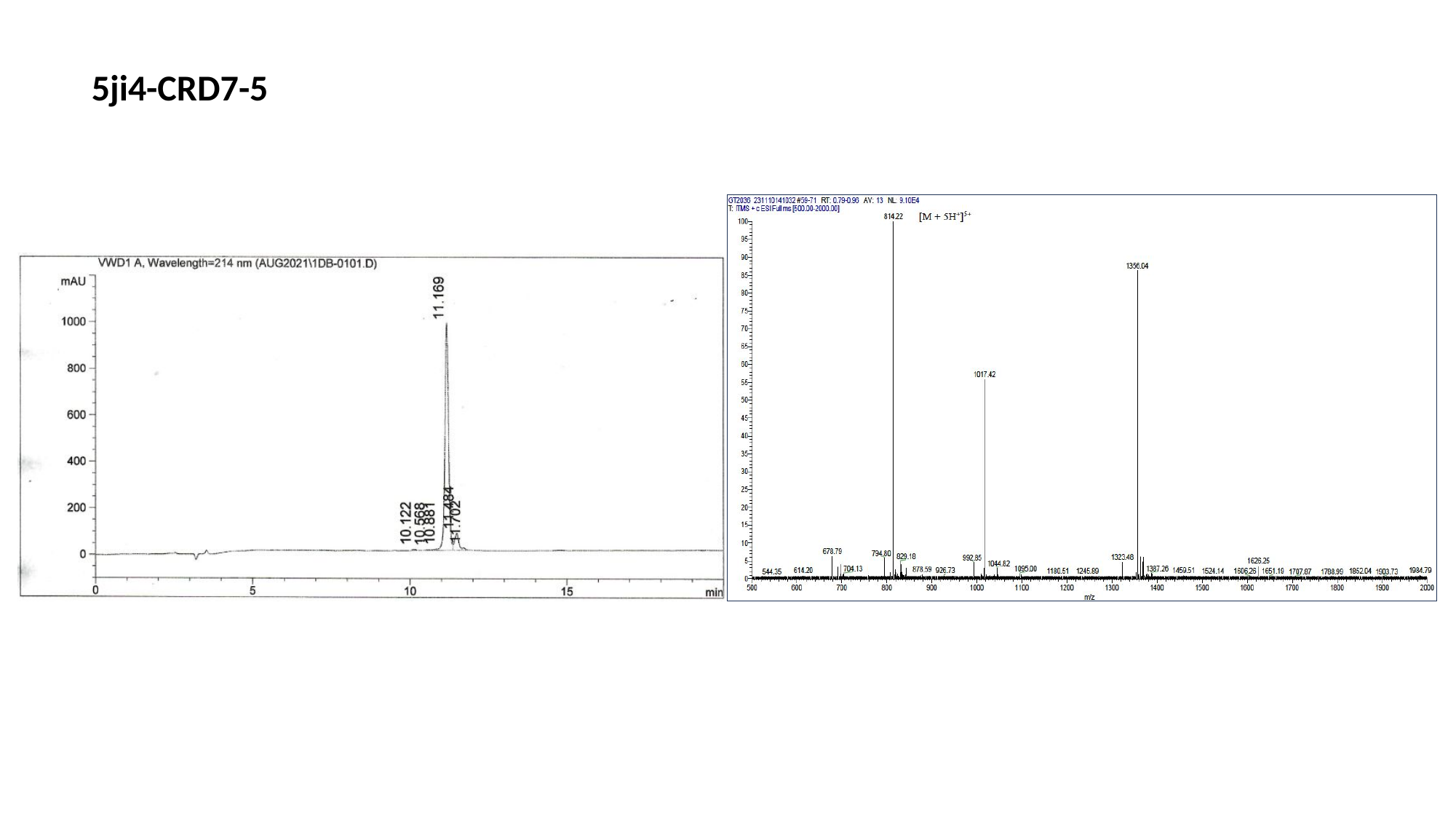

5ji4-CRD7-5

## Slide 16
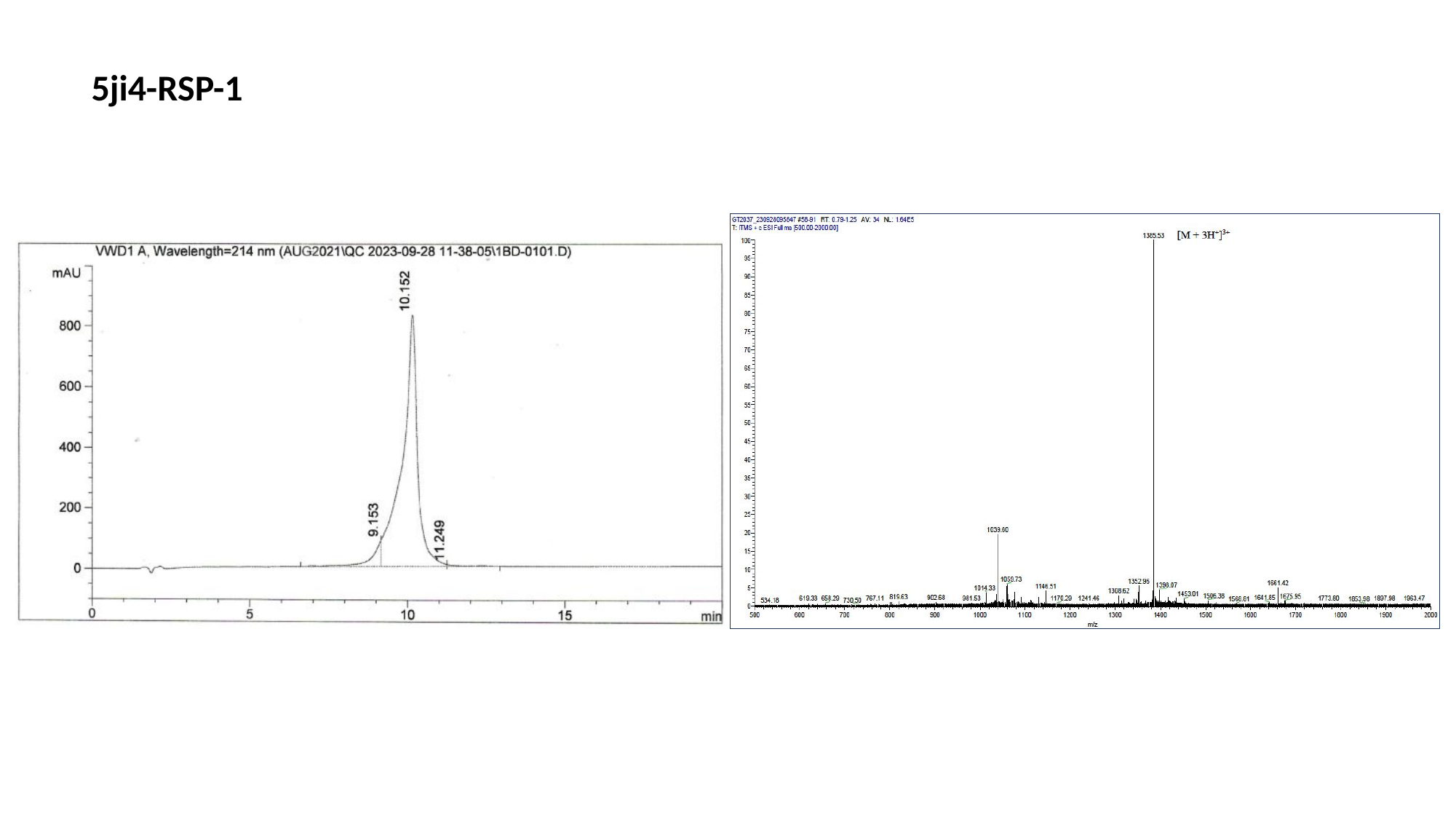

5ji4-RSP-1

## Slide 17
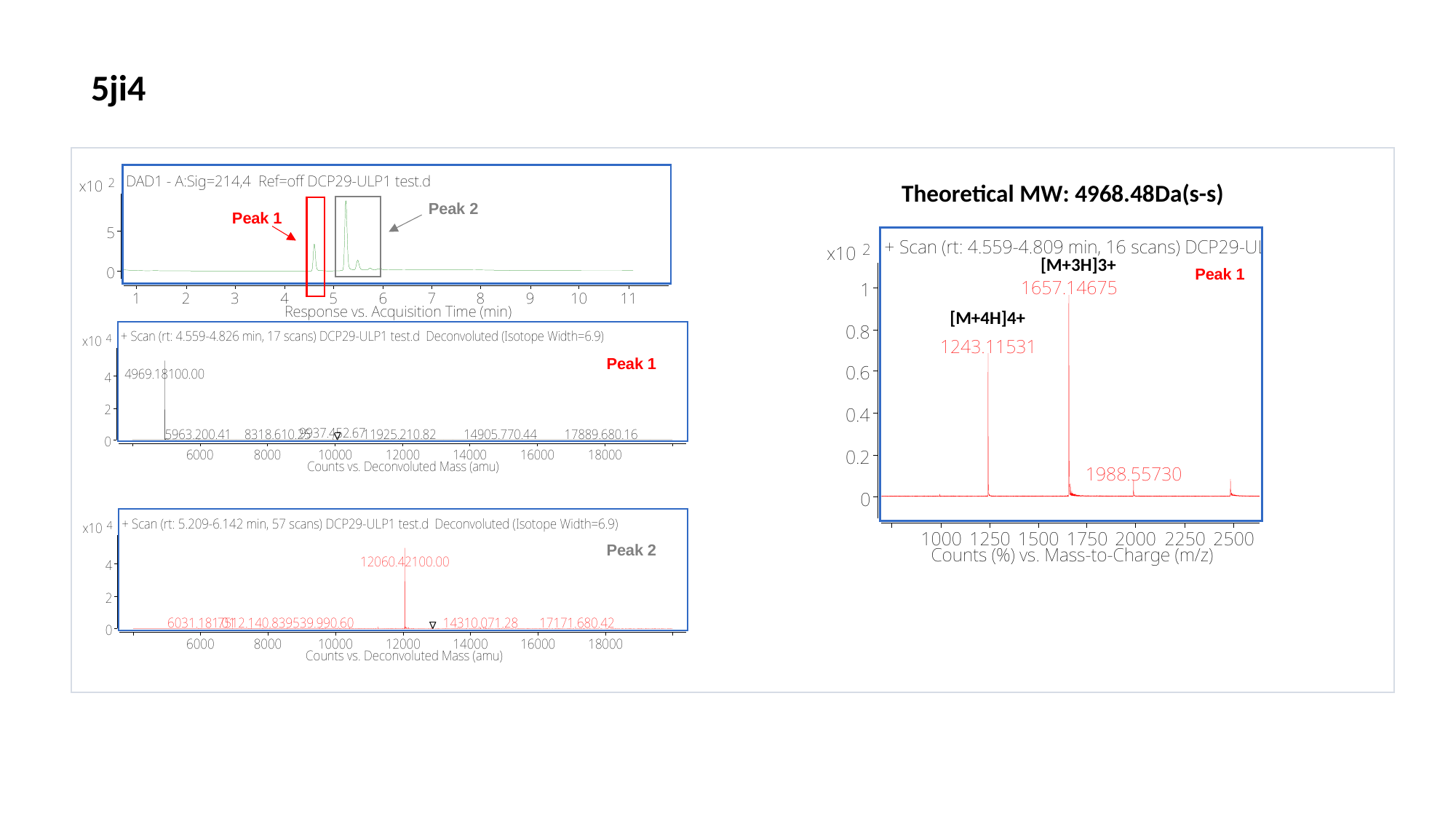

5ji4
Peak 2
Peak 1
Theoretical MW: 4968.48Da(s-s)
[M+3H]3+
Peak 1
[M+4H]4+
Peak 1
Peak 2

## Slide 18
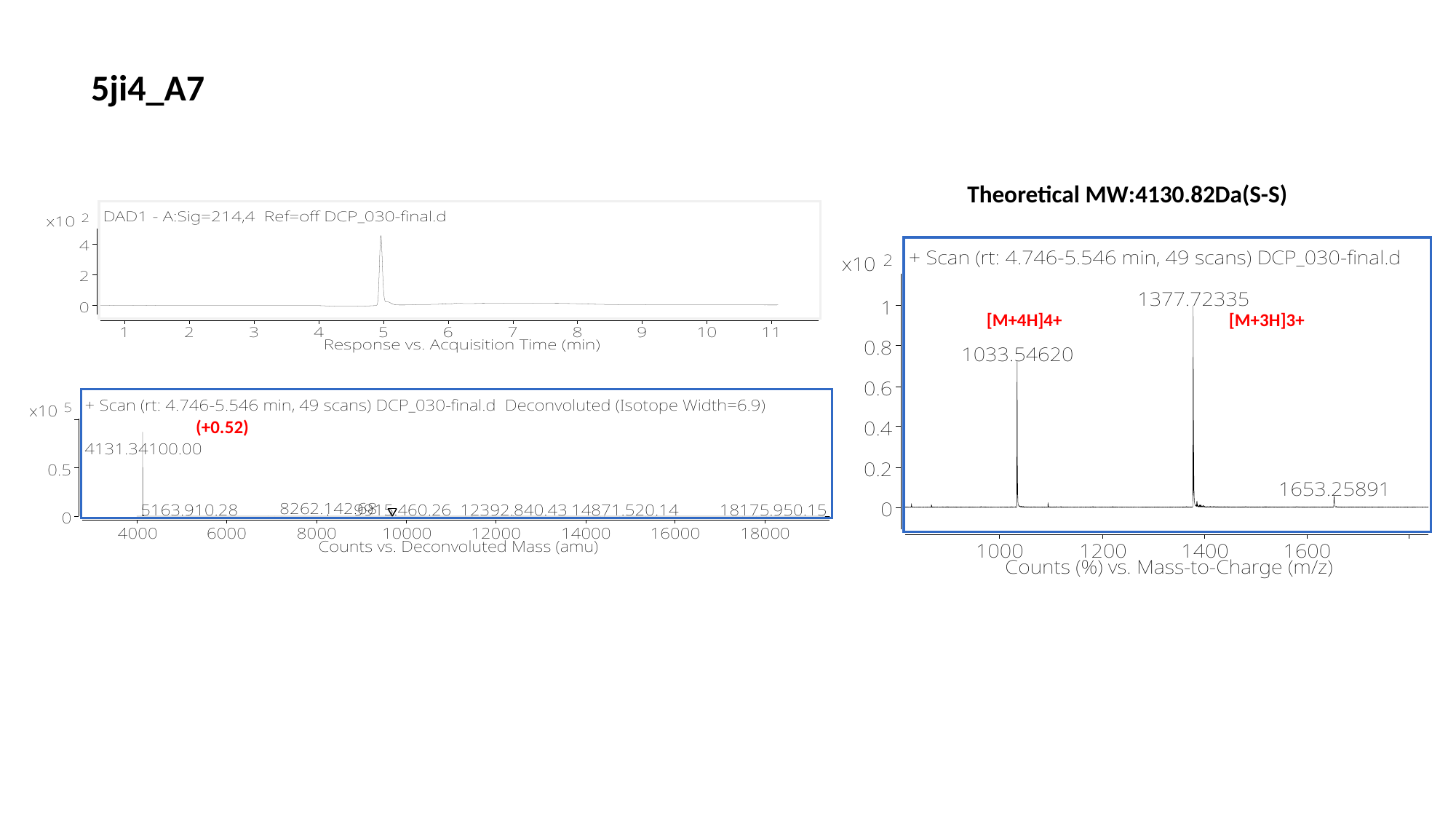

5ji4_A7
Theoretical MW:4130.82Da(S-S)
[M+3H]3+
[M+4H]4+
(+0.52)

## Slide 19
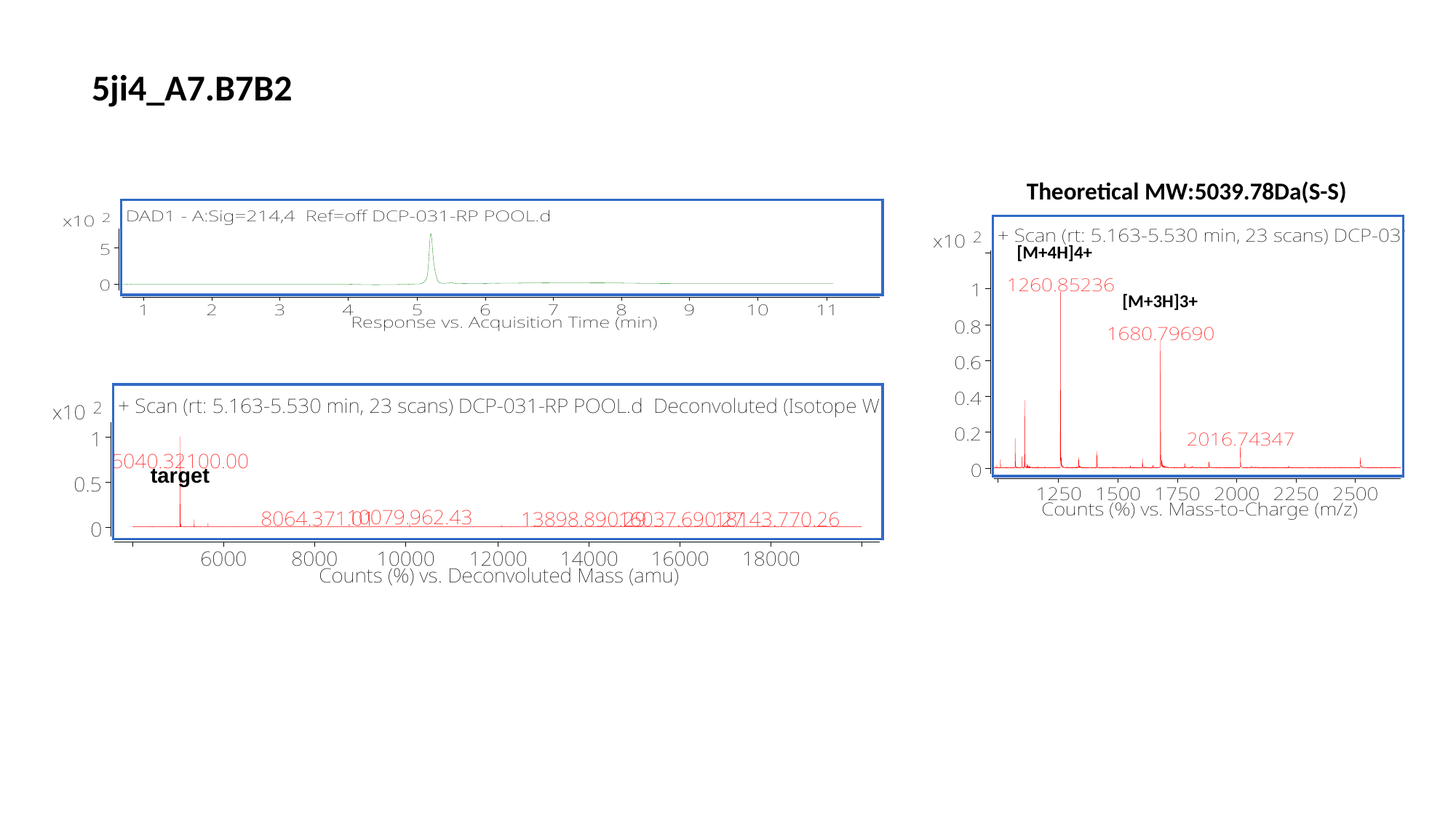

5ji4_A7.B7B2
Theoretical MW:5039.78Da(S-S)
[M+4H]4+
[M+3H]3+
target

## Slide 20
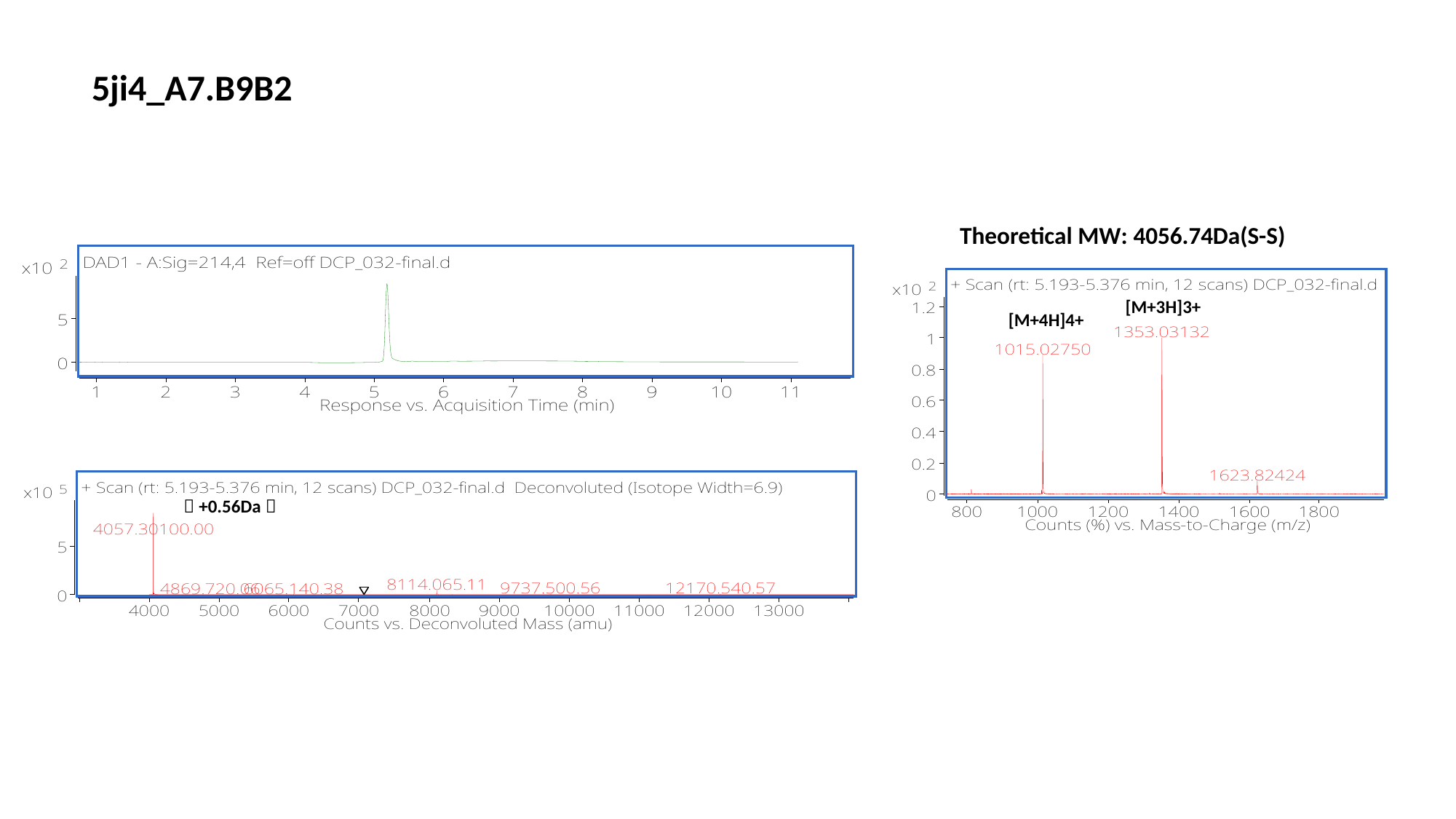

5ji4_A7.B9B2
Theoretical MW: 4056.74Da(S-S)
[M+3H]3+
[M+3H]3+
[M+4H]4+
[M+4H]4+
(+0.46Da)
（+0.56Da）

## Slide 21
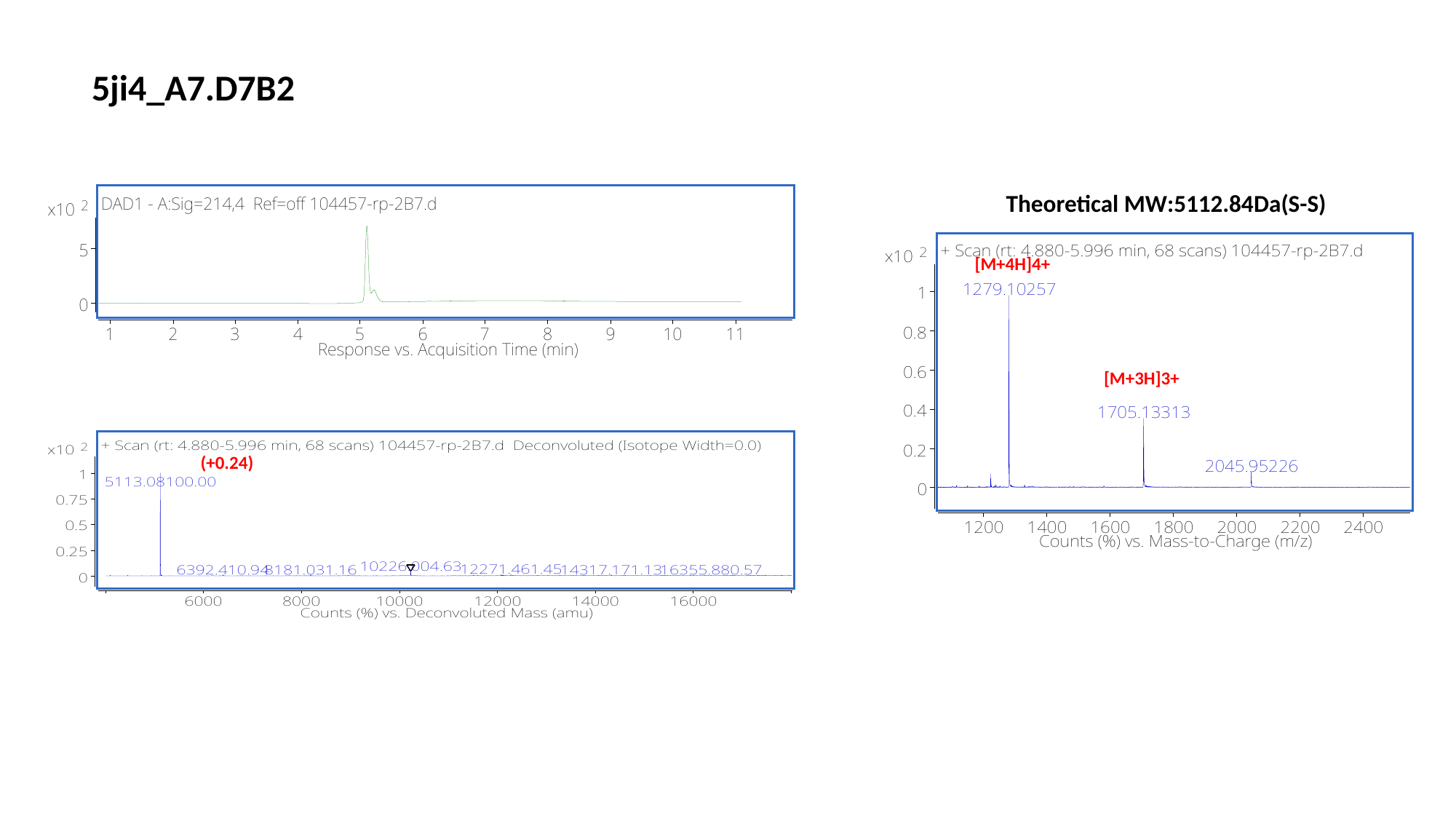

5ji4_A7.D7B2
Theoretical MW:5112.84Da(S-S)
[M+4H]4+
[M+3H]3+
(+0.24)

## Slide 22
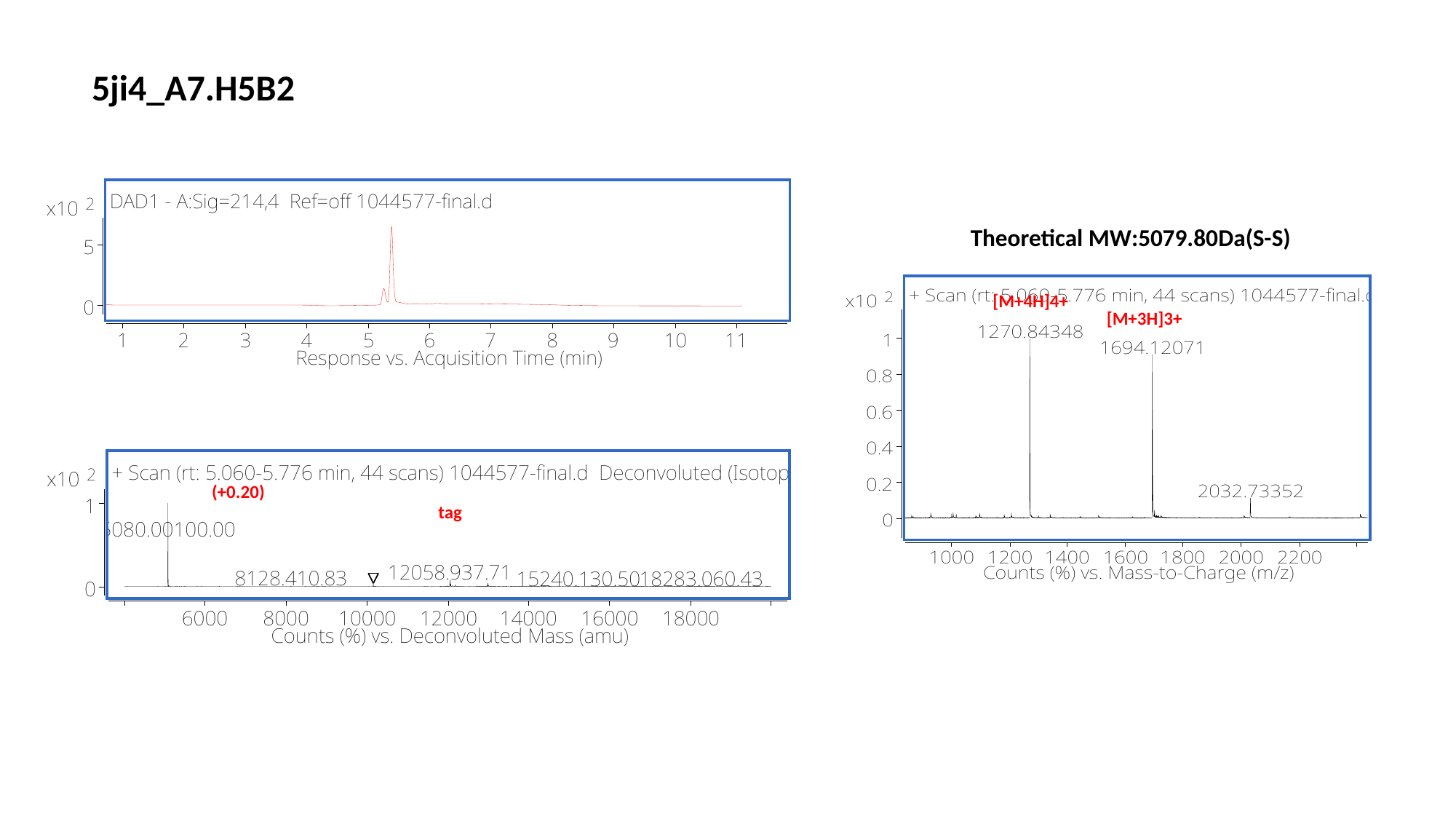

5ji4_A7.H5B2
Theoretical MW:5079.80Da(S-S)
[M+4H]4+
[M+3H]3+
(+0.20)
tag

## Slide 23
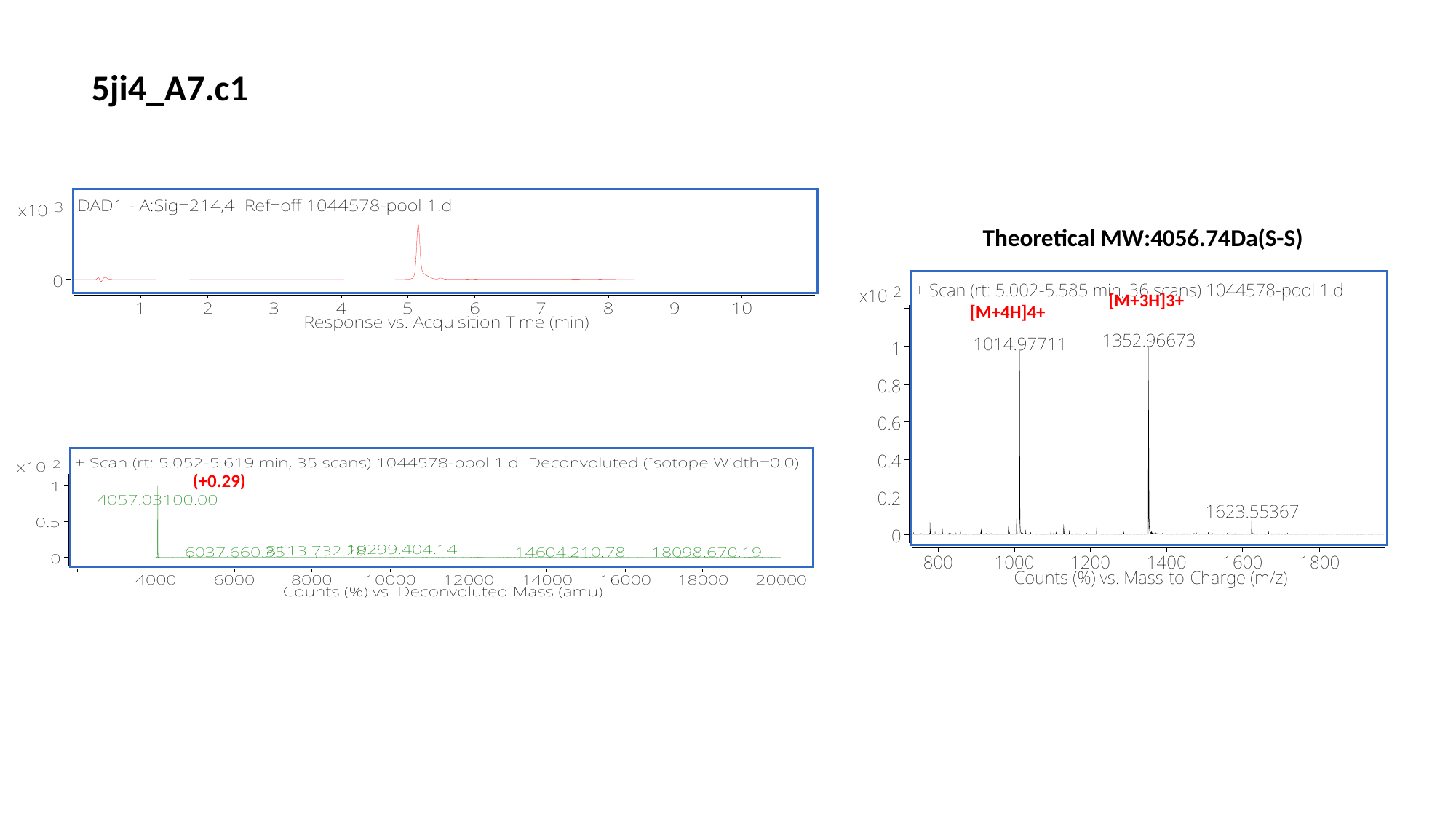

5ji4_A7.c1
Theoretical MW:4056.74Da(S-S)
[M+3H]3+
[M+4H]4+
(+0.29)
