# Supplementary material for: Rapid prediction of key residues for foldability by machine learning model enables the design of highly functional libraries with hyperstable constrained peptide scaffolds
Source: PLoS Comput Biol. 2024 Nov 18;20(11):e1012609. doi: 10.1371/journal.pcbi.1012609 (PMC11611271; doi:10.1371/journal.pcbi.1012609)
Supplement: Attachment — Submitted filename: 092524ReviewDetail.docx [file pcbi.1012609.s011.docx]

Dear Editor:

Thank you very much for your review. We appreciate your consideration for re-submission after revision.

Following is the response from us to the two reviewers’ comments. The revision in the manuscript has been indicated, which is highlighted in yellow. The line numbers refer to the numbers in the clean version without tracked changes. Our responses are in bold italic font.

Two versions of manuscript are re-submitted: one with tracked changes on top of the previous submission and one clean version without tracked changes. New Figures and supporting materials are also uploaded accordingly.

Best Regards,

Yingnan Zhang and Andrew Chang

***********************

Reviewer's Responses to Questions

**Comments to the Authors:
Please note here if the review is uploaded as an attachment.**

Reviewer #1: Disulfide-constrained peptides represent a promising peptide modality for drug design and therapeutic applications. However, their limited foldability poses challenges for library design, which is essential for discovering functional peptides. Cai and colleagues hypothesized that specific sequence patterns within these peptide scaffolds play a critical role in their spontaneous folding into stable topologies. Consequently, these sequences should be avoided during randomization in the initial library design. To create highly diverse disulfide-constrained peptide (DCP) libraries while preserving the inherent foldability of each scaffold, the researchers generated a large-scale dataset using yeast surface display combined with shotgun alanine scanning experiments. This dataset was used to train a machine-learning model based on a technique employed in natural language understanding. The authors validated the model through experimental methods, demonstrating that it can accurately predict the foldability of peptides across a broad range of sequences and identify residues critical for foldability. Building on these findings, the authors designed a new peptide library based on a de novo-designed disulfide-constrained peptide scaffold optimized for enhanced folding efficiency. Screening this library against various protein targets yielded peptides with good binding affinity. This study is likely to interest researchers in the fields of peptide design and drug discovery. I recommend the publication of this work, pending the resolution of the following issues.

1. It is unclear whether the BiFC assay can distinguish between natively-folded peptide and the disulfide-mispaired fold. Thus, at least two or three model sequences with different foldability should be selected to validate the BiFC assay.

***We have added more controls for BiFC assay and shown results in new S1-Figures B. The new control includes: one peptide with all Cys mutated to Ala for 5ji4 to enable unfolded or partially folded peptide by deleting disulfide constrain; a linear peptide with peptide tags in tandem plus GS linker with similar length as 5ji4 and a peptide with all key residues for foldability mutated to Ala. As shown in S1-Figure2B and 2C, all three negative peptides showed significantly lower mean fluorescence than the positive, well-folded 5ji4. This result exemplified that BiFC system could distinguish folded vs unfold or linear peptides. We have included this result in new S1-Figures as S1-Figure2B and 2C and added the description in main text from line166-172.***

2. HPLC chromatograms showing the oxidative folding of the peptide binders identified from library screening should be provided.

***All LC-MS data for all HCP mentioned in this study are provided in supporting material S3_peptideLCMS.pptx***

3. It would be interesting to experimentally characterize or computationally predict the structures of the identified peptide binders. A comparison between the structures of the identified peptides and that of the precursor scaffold should be conducted and discussed.

***Unfortunately, we did not solve the structure for any of the mutated HCPs reported in this study. An AF2-predicted structure of HtrA1-A7 superimposed with its precursor scaffold 5ji4 is shown in S1-Figures as S1-Figure5. The disulfide connectivity appears to be maintained in the model of Htra1-A7 and the overall structures are superimposable with an RMSD value of 0.750 Å. However, we notice that the C4-C19 disulfide is actually not formed in the AF2 model, although the side chains of the two Cys residues are in close proximity (2.4Å). The main difference lies in the region that had been mutated (residues 5-14) forming an antiparallel beta: the loop connecting the first and the second β-strand adopts a different conformation, the main chain being displaced by about 4Å from its original position as seen in 5ji4. . The residues that had been identified as untouchable (residue 15-27) are mainly involved in alpha helix formation and were kept unchanged in HtrA1-A7, hence the excellent superposition of this region (RMSD of 0.257 Å). Therefore, for 5ji4 the sequences that are responsible for alpha-helix formation are the determinants for foldability, consistent with the high ES in Ala-scan experiments, as well as the high score from our ML model prediction. We include this description in “Results/Identification of functional inhibitors of the protease HtrA1” in line 510-519.***

Reviewer #2: This work aims to improve peptide foldability for high-throughput yeast or phage display by constructing a sequence foldability database and training a BERT-based classification model. Why the model and data are of some value, peptide structures can now be predicted using AF2 or similar methods and peptide sequences can be designed using inverse programs. Large scale display approaches are of less value and should be combined with these kind of predictive models.

***In our previous in-house work with the DCP platform (1), we noticed that close to 50% of the identified lead peptides, whose X-ray crystal structures had been determined, adopted a totally different disulfide connectivity and conformation from their original parent scaffolds, while still maintaining high stability. AF2 or similar methods such as ProteinMPNN completely failed in predicting these novel peptide structures, because the predictions are based on the parent disulfide connectivity. Even when the parent scaffold was maintained, as in HtrA1-A7, the AF2 model lacked precision in regard to disulfide formation (see comment above). Therefore, it is our opinion that the current structure prediction models lack the accuracy required for dealing with the structural diversity of peptides encoded by our libraries. Since the examples I mentioned here were proprietary information and cannot be disclosed to public, we did not discuss these results in this manuscript.***

***The main purpose of this study was to predict sequences with good foldability, independent of whether the original fold was maintained or not. In fact, it seemed important to us to include all variants with different disulfide connectivity, as long as they have good foldability. Consequently, incorporation of these sequences in our ML models significantly expanded the library’s conformational diversity and improved library effectiveness. Therefore, we decided to pursue a “structure-agnostic” approach that is solely based on primary sequence data derived from large-scale display experiments. We now discuss this point in the Discussion section line 541-549, and elaborate this point further in line 570-595.***

***To emphasize the significance of the study, we also added two paragraphs in “Introduction”, line 94-97 and 103-109, to recapitulate the above points.***

Some specific questions:
1. One of the key postulates in this work is that any library member capable of successful surface display on yeast cells must fold into a well-defined 3D structure, which may be true for well folded proteins, but for “peptides” that are normally flexible, it might be very different. An issue here is that what is the definition of “peptide” that the authors referred to. For common understandings, peptides usually do not have well folded structures. However, during the validation of generalizability, the protein with PDB code 1BH4, which is primarily composed of loops with various conformations, was selected. Does this conflict with the postulate? Additionally, the ES scores are near zero for both experimental results and predicted values. Does this imply that no residue is crucial for folding?

***In this study, we focused on poly peptides with sequences shorter than 47 amino acids. These are actually mini-proteins with well-defined 3D structures maintained by disulfide constrain. We call these mini-proteins as “peptide” solely because they are within the size range for chemical synthesis. Yeast display method can distinguish folded vs unfolded mini-proteins for such size. In order to validate this point, we have added one more experiment to include a few controls for yeast surface display. As shown in S1-figure2A and 2C, a well-folded HCP (‘positive’ 5JI4) has significantly higher display level than peptide with all Cys muted to Ala (5ji4CtoA), or a peptide with all key residues for foldability mutated to Ala (5ji4N) as well as a linear peptide with similar length (SFS). The FACS sorting could effectively enrich the well-folded population with such signal window. We described this result in the text in line143-156 in session of “Shotgun Alanine scan as an effective method to generate SAR information between sequences and foldability”.***

***We chose 1BH4 for generalization test because its sequence is remotely related to HCPs that we used to generate training data and, unlike HCPs, it contains no well-defined secondary structures. We originally postulate that certain intrinsic sequence that governing the foldability should enable the proper disulfide connectivity. It turned out that almost no critical residues can be identified via both experiment and ML prediction. The folding, or rather the disulfide formation might solely rely on the position of Cysteine instead of specific sequences. We noticed a few other scaffolds behaves like this. This phenomenon does not contradict to our postulate, instead, it re-enforced our observation that for scaffolds with well-defined secondary structures, certain residues are critical for folding, whereas for scaffolds with mostly unstructured loops, almost all residues are amenable as long as the positions of Cys are maintained. The effective libraries can be designed accordingly. In fact, the most successful library in our previous study(1), EETI-II, is similar to 1BH4. We observed that almost the full sequence is amenable and the library were highly effective.***

2. Sequence generation and diversity. How many sequences are generated from the shotgun alanine scanning? Additionally, what is the sequence diversity?

***We have reported this information in S4-Table2 and described in main text in “Results/ Data generation for machine learning model with HCP scaffolds” in line 231-234.***

3. Detailed residue contribution. In Figures 1A, 2, and 4A, the ES scores of a continuous non-touchable peptide segment are close. How do you distinguish the detailed contribution of each residue?

***The definition of non-touchable residues is relative to the other residues within the same scaffold. It is important to note that ES scores are not directly comparable across different scaffolds, as the importance of each residue is influenced by the surrounding residues within the same scaffold. Therefore, comparing residues within a single scaffold is more meaningful than across different ones.***

***For each peptide, we define 'non-touchable' residues as those with ES values equal to or greater than the average ES value within the scaffold, as shown in S1-Figure5. Based on this definition, some scaffolds exhibit continuous regions where most residues have ES values at or above the average, which is biologically plausible, as a continuous region may have a more significant impact on foldability than isolated point mutations.***

4. Model adjustment. To achieve a higher F1-score, the model was adjusted from a ternary classification model to a binary classification model. Why did you adjust the ternary classification model instead of retraining the binary classification model from the original ratio data?

***Thank you for the insightful question. We initially trained a binary classifier using labels for High and Low foldability to predict ES scores. However, this approach resulted in suboptimal performance, with predictions close to random, particularly in the ES score prediction task. This likely stems from the significant ambiguity in classifying sequences strictly as 'High' or 'Low' foldability, which caused the model to struggle in capturing the patterns associated with 'Mid' or ambiguous foldability levels.***

***By incorporating a Mid-foldability class, the model was able to predict ES scores more accurately, indicating that it could better learn the true patterns distinguishing High foldability from Low foldability. As described in the main manuscript, to fairly assess this classification model, we adjusted the F1 score to account for the inclusion of this Mid-foldability class.***

***We believe that the model's precision and accuracy could be significantly improved by refining the classification beyond just High, Mid, and Low foldability. However, achieving this would require the collection of finer bins from a FACS experiment across different levels of yeast display expression, as well as deeper sequencing to generate enough reads to train the model to recognize the subtleties in foldability at various levels. This is an excellent point and could be an interesting direction for future research.***

5. Visualization of Figure 2 data. The data in Figure 2 are too dense and the values are very similar. Please plot the model's prediction results as a bar chart and adjust the y-axis accordingly.

***We have made a new Figure 2 in a logo format (bar plot) and put the table with the actual numbers into Supporting Materials as S1-Figure 3. We corrected the figure reference citing in the main text accordingly as well as the figure legend for Figure 2.***

**Have the authors made all data and (if applicable) computational code underlying the findings in their manuscript fully available?**
The [PLOS Data policy](https://c05y1x9s.r.us-east-2.awstrack.me/L0/https:%2F%2Fjournals.plos.org%2Fploscompbiol%2Fs%2Fmaterials-and-software-sharing/1/010f019146124c4a-cb00e911-8f5c-4e61-8c27-da93d1a7f51b-000000/Qd4eU2vujFNScJcYz5LbQ1m3jac=171) requires authors to make all data and code underlying the findings described in their manuscript fully available without restriction, with rare exception (please refer to the Data Availability Statement in the manuscript PDF file). The data and code should be provided as part of the manuscript or its supporting information, or deposited to a public repository. For example, in addition to summary statistics, the data points behind means, medians and variance measures should be available. If there are restrictions on publicly sharing data or code —e.g. participant privacy or use of data from a third party—those must be specified.

Reviewer #1: Yes

Reviewer #2: None

***We have provided all data that used to generate graphs in the supported materials.***

PLOS authors have the option to publish the peer review history of their article ([what does this mean?](https://c05y1x9s.r.us-east-2.awstrack.me/L0/https:%2F%2Fjournals.plos.org%2Fploscompbiol%2Fs%2Feditorial-and-peer-review-process%23loc-peer-review-history/1/010f019146124c4a-cb00e911-8f5c-4e61-8c27-da93d1a7f51b-000000/2NZFhAZy8NtwqGm3r7lk0PZlxeg=171)). If published, this will include your full peer review and any attached files.


If you choose “no”, your identity will remain anonymous but your review may still be made public.


**Do you want your identity to be public for this peer review?** For information about this choice, including consent withdrawal, please see our [Privacy Policy](https://c05y1x9s.r.us-east-2.awstrack.me/L0/https:%2F%2Fwww.plos.org%2Fprivacy-policy/1/010f019146124c4a-cb00e911-8f5c-4e61-8c27-da93d1a7f51b-000000/UUkW9L1dd1d9ybfnW0yQuFl9tHM=171).

Reviewer #1: No

Reviewer #2: No

Figure Files:

While revising your submission, please upload your figure files to the Preflight Analysis and Conversion Engine (PACE) digital diagnostic tool, [https://pacev2.apexcovantage.com](https://c05y1x9s.r.us-east-2.awstrack.me/L0/https:%2F%2Fpacev2.apexcovantage.com%2F/1/010f019146124c4a-cb00e911-8f5c-4e61-8c27-da93d1a7f51b-000000/nUoTCf4XNFgexCsceKPRiTq04xk=171). PACE helps ensure that figures meet PLOS requirements. To use PACE, you must first register as a user. Then, login and navigate to the UPLOAD tab, where you will find detailed instructions on how to use the tool. If you encounter any issues or have any questions when using PACE, please email us at [figures@plos.org](mailto:figures@plos.org).

Data Requirements:

Please note that, as a condition of publication, PLOS' data policy requires that you make available all data used to draw the conclusions outlined in your manuscript. Data must be deposited in an appropriate repository, included within the body of the manuscript, or uploaded as supporting information. This includes all numerical values that were used to generate graphs, histograms etc.. For an example in PLOS Biology see here: <http://www.plosbiology.org/article/info%3Adoi%2F10.1371%2Fjournal.pbio.1001908#s5>.

Reproducibility:

To enhance the reproducibility of your results, we recommend that you deposit your laboratory protocols in [protocols.io](http://protocols.io/), where a protocol can be assigned its own identifier (DOI) such that it can be cited independently in the future. Additionally, PLOS ONE offers an option to publish peer-reviewed clinical study protocols. Read more information on sharing protocols at [https://plos.org/protocols?utm_medium=editorial-email&utm_source=authorletters&utm_campaign=protocols](https://c05y1x9s.r.us-east-2.awstrack.me/L0/https:%2F%2Fplos.org%2Fprotocols%3Futm_medium=editorial-email%26utm_source=authorletters%26utm_campaign=protocols/1/010f019146124c4a-cb00e911-8f5c-4e61-8c27-da93d1a7f51b-000000/js8nCxn0fK0l6FpFgabSvqZ2rxA=171)

1.	Zhou L, Cai F, Li Y, Gao X, Wei Y, Fedorova A, et al. Disulfide-constrained peptide scaffolds enable a robust peptide-therapeutic discovery platform. PLoS One. 2024;19(3):e0300135.
